# MG-SpaIR: Multi-Grade Sparse-Guided Implicit Representation for Training-Data-Free Image Restoration

**DOI:** 10.1007/s10851-026-01329-2

**Published:** 2026-07-30

**Authors:** Jianmin Liao, Lei Huang, Ronglong Fang, Ashley Prater-Bennette, Lixin Shen, Yuesheng Xu

**Affiliations:** 1 Department of Mathematics, Syracuse University, 215 Carnegie Building, Syracuse, NY 13210 USA; 2 Department of Mathematics & Statistics, Old Dominion University, 2300 Engineering & Computational Sciences Building, Norfolk, VA 23529 USA; 3 Department of Medical Physical, Memorial Sloan Kettering Cancer Center, 1250 First Avenue, New York, NY 10065 USA; 4 Air Force Research Laboratory, 525 Brooks Road, Rome, NY 13441 USA

**Keywords:** Image restoration, Training-data-free, Implicit neural representation, Sparse regularization, Multi-grade deep learning

## Abstract

MG-SpaIR is a training-data-free framework for restoring a clean image from a single observation corrupted by a mixture of blur, downsampling, noise, and missing pixels. Building on implicit neural representations (INRs), we introduce a multi-grade residual hierarchy that progressively refines the reconstruction from low to high spatial frequencies across grades, improving representational fidelity and mitigating spectral limitations. To stabilize reconstruction optimization and suppress INR-induced artifacts, we further propose an explicit sparse proximal regularization (e.g., $\ell _0$ type) applied directly in the high-resolution image domain, which discourages spurious high-frequency patterns while preserving sharp structures. The resulting optimization is solved efficiently via a multi-grade proximal alternating scheme, and we establish convergence guarantees for the associated updates under standard regularity conditions. Experiments on mixed-degradation benchmarks demonstrate that MG-SpaIR consistently outperforms strong training-data-free baselines such as Deep Image Prior, providing a stable, interpretable, and data-efficient alternative to conventional learning-based restoration methods.

## Introduction

We study the restoration of a clean image from a single observation corrupted by a mixture of degradations—blur, downsampling, noise, and missing pixels—reflecting common failure modes in real imaging systems. Because many clean images can explain the same observed input, image restoration is inherently ill-posed, making effective priors essential for accurate recovery.

Classical restoration methods rely on explicit priors, most notably sparsity-based regularization such as total variation [1] and non-local self-similarity as in BM3D [2]. These methods operate directly on pixel-based image representations and therefore rely on explicit priors, rather than exploiting the implicit prior afforded by the neural architecture itself.

Neural networks have been explored as implicit image priors for their ability to capture natural image statistics [3]. Implicit neural representations (INRs) [4–10] model images as continuous coordinate-to-color mappings, naturally recovering missing pixels and fine details, making them effective for training-data-free tasks like super-resolution and inpainting.

Despite their appeal, standard INRs for restoration face two fundamental challenges. First, the inherent spectral bias [10, 11] impedes the accurate representation of high-frequency structures, leading to blurry textures and poor detail recovery. Second, reliance solely on the implicit architectural prior often destabilizes the training-data-free optimization, injecting spurious high-frequency artifacts (e.g., ringing, checkerboards). This artifact tendency is evident in prior works (e.g., Fig. 7 in [6], Supp. Fig. 8 of [4]) and is confirmed by our experiments (Figs. 8 and 7). To the best of our knowledge, we are among the first to explicitly identify the tendency of INRs to generate artifacts and to propose an explicit regularization solution.

To address this challenge, we introduce multi-grade sparse-guided implicit representation (MG-SpaIR) for image representation. MG-SpaIR combines multi-grade deep learning with sparse regularization to improve image reconstruction quality. Specifically, the multi-grade learning strategy, introduced in [12], decomposes the end-to-end optimization of a deep network into a sequence of smaller subproblems, referred to as grades. At each grade, a shallow subnetwork is trained to fit the residuals generated by the previous grades. The newly trained subnetwork is then composed with the previously learned subnetworks, whose parameters remain fixed and serve as adaptive feature extractors or basis functions. This progressive learning strategy has been shown to mitigate the spectral bias of neural networks and improve optimization stability [13, 14]. In addition, natural images often exhibit sparse structures in suitable transform domains, such as total variation and the discrete cosine transform, whereas random noise typically remains unstructured. Exploiting this property, sparse regularization has been extensively used in traditional image reconstruction and inverse problems to suppress noise while preserving important image features. Motivated by its success in these settings, we incorporate sparse regularization into the multi-grade implicit representation framework to improve reconstruction quality and reduce high-frequency artifacts. Our main contributions are summarized as follows:

- *Multi-grade INR to mitigate spectral limitations.* We propose a residual hierarchy that progressively learns high-frequency content across grades, improving detail recovery while remaining compute-efficient.
- *Sparse proximal regularization for artifact suppression.* We introduce an explicit $\ell _0$-type sparse proximal prior in the restored image domain (i.e., the high-resolution target domain for super-resolution) to stabilize reconstruction and suppress INR-induced artifacts.
- *Convergence analysis.* We provide convergence guarantees for the proposed optimization scheme under standard regularity assumptions.
- *Empirical validation.* We demonstrate consistent improvements over strong training-data-free baselines (e.g., DIP) across mixed-degradation benchmarks.

**Fig. 1:**
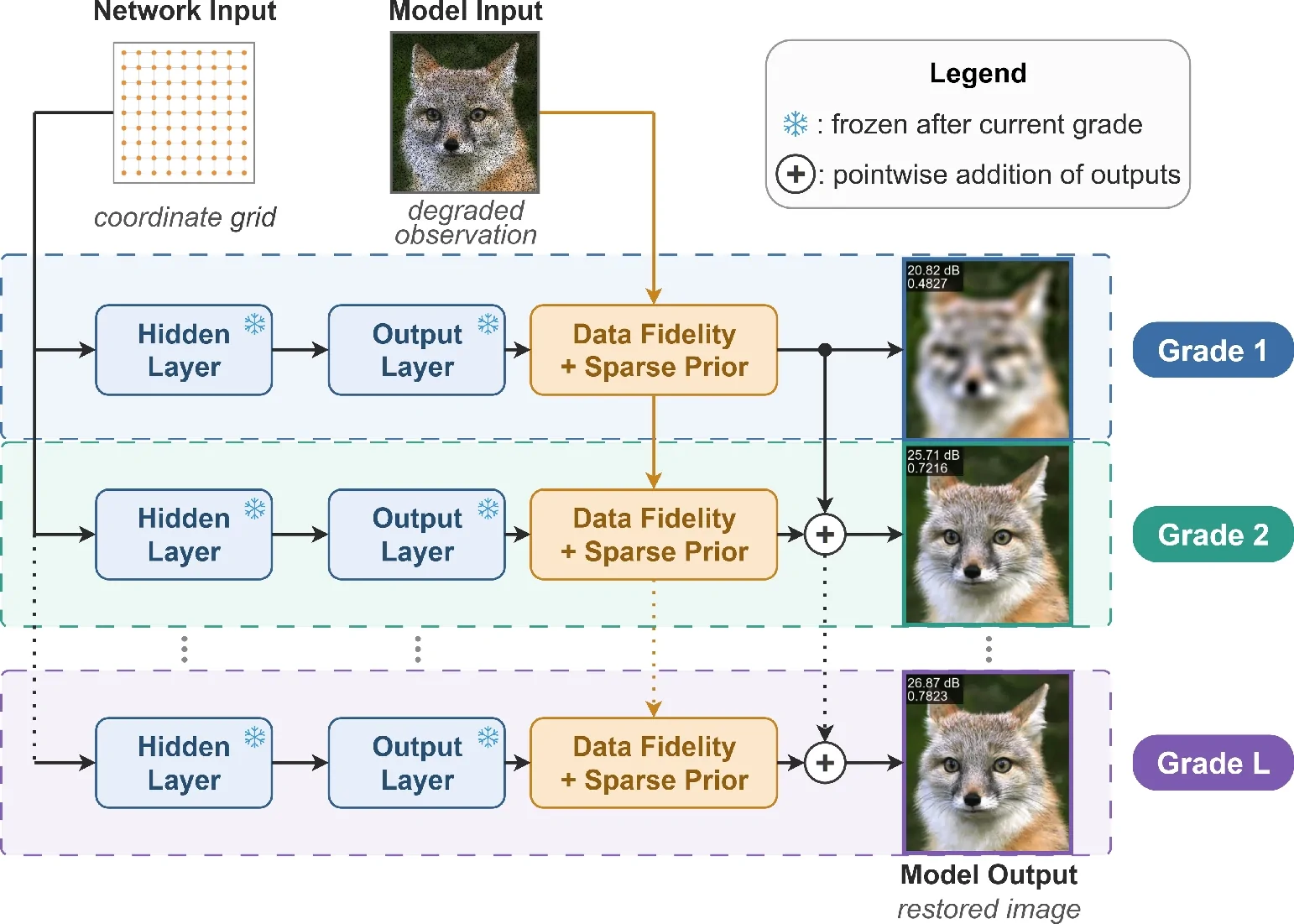
Architecture Overview. The restoration model is optimized against the observed input, while the network input is a uniform coordination grid over $[-1,1]^2$. The restored image is implicitly encoded in the neural network parameters and guided by data fidelity and sparse prior during optimization. Training proceeds in a sequence of grades (Grade 1 through Grade *L*, where *L* is an arbitrary, user-specified number indicated by the ellipsis): At each grade, the parameters from all previous grades are frozen, and only the current grade is updated. Each new grade learns from the residual of the previous reconstruction, progressively refining image details. The output of the final grade produces the restored image. The circled “+” denotes the additive aggregation of the (frozen) previous grades’ outputs with the current grade’s output

An overview of the proposed multi-grade implicit representation framework is illustrated in Fig. 1,^1^. Each grade corresponds to a shallow INR optimized directly on the observed input. Through progressive refinement, the network’s representational ability improves across grades, capturing fine structures and recovering missing details. As shown in the figure, sparse proximal regularization is applied at each grade in the high-resolution image domain to guide the optimization toward stable and faithful reconstructions.

The remainder of this paper is organized as follows. Section 2 reviews related work on implicit neural representations and image restoration. Section 3 presents the proposed model formulation, including the multi-grade strategy, and details the MG-SpaIR regularization and the optimization algorithm for solving the resulting objective. Section 4 provides convergence analysis of the proposed scheme. Section 5 reports experimental results and ablation studies. Finally, Sect. 6 concludes the paper and discusses limitations and future directions.

## Related Work

*Classical explicit priors.*  Classical restoration methods rely on explicit priors to constrain the solution space. Sparsity-based constraints, such as $\ell _1$/TV [1], $\ell _0$ [15–18], and non-convex surrogates (SCAD [19], MCP (minimax concave penalty) [20]), are typically solved via proximal algorithms. Non-local priors (e.g., NLM [21], BM3D [2]) utilize self-similarity to suppress noise but often incur prohibitive computational costs at high resolutions. These methods operate directly on pixel-based image representations and therefore rely on explicit priors, rather than exploiting the implicit prior afforded by the neural architecture itself.

*Data-driven versus implicit neural priors.*  Deep learning approaches have largely superseded classical methods, yet they face the trade-off between fidelity and perceptual quality. Supervised models like SwinIR [22] and Restormer [23] achieve state-of-the-art perceptual quality by learning statistical priors from massive datasets. However, this dependence often leads to *hallucinated textures*—details that appear realistic but do not exist in the ground truth [24, 25], rendering them unreliable for fidelity-critical applications. Conversely, implicit neural priors like DIP [3] avoid dataset bias by exploiting the inductive bias of network architectures. However, without explicit constraints, the optimization landscape of these purely implicit methods is often treacherous, leading to instability and high-frequency artifacts as the network overfits to noise.

*Implicit neural representations.*  Implicit neural representations (INRs) model continuous signals via neural networks and are widely applied in geometry and imaging. Early works such as DeepSDF [26] and Occupancy Networks [27] established implicit 3D modeling. Subsequent advances addressed spectral bias through oscillatory bases (SIREN [4], WIRE [6], MFN [28], Residual MFN [29]), and input encodings [5, 11]. In this work, we primarily adopt SIREN and WIRE as INR backbones. Both are coordinate-based MLPs that map spatial coordinates to signal values, and their activation functions are designed to better capture high-frequency signal components than standard ReLU-based MLPs. Specifically, SIREN uses sinusoidal activations, while WIRE uses Gabor wavelet-based activations. The detailed layer functions of these backbones are provided in Sect. A.1. Further control over frequency and scale was introduced via progressive or band-limited encodings [30, 31] and adaptive multi-scale [32] or meta-learned frameworks [33].

*Spectral bias of neural networks.*  Previous studies [13, 14, 34, 35] have shown that neural networks preferentially learn low-frequency components while underrepresenting high-frequency details. This phenomenon, known as *spectral bias*, limits their performance in applications requiring fine high-frequency reconstruction, such as image restoration.

*INRs for image restoration.*  Existing INR-based methods incorporating explicit priors for denoising or inpainting often rely on non-local self-similarity [36] or TV regularization [37]. In contrast, we introduce (i) a multi-grade strategy to mitigate spectral bias and enhance the INR’s representational accuracy, and (ii) an explicit sparsity prior in the *high-resolution* image domain, extending beyond TV/$\ell _1$ to $\ell _0$ and MCP regularizations to directly suppress high-resolution artifacts and noise.

## Proposed Model and Algorithm

In this section, we present the MG-SpaIR optimization model. We first formulate a unified image restoration setting, then describe our multi-grade strategy for enhancing image representation, and finally introduce our restoration model along with its optimization algorithm.

Let $\textbf{Y} \in \mathbb {R}^{H \times W \times C}$ denote a latent clean image with height *H*, width *W*, and *C* channels, and let $\tilde{\textbf{Y}} \in \mathbb {R}^{\frac{H}{s} \times \frac{W}{s} \times C}$ denote the corresponding observed input, where $s \in \mathbb {N}$ is the downsampling factor and $\frac{H}{s}, \frac{W}{s} \in \mathbb {N}$. We adopt a unified degradation model expressed as:

1 $$\begin{aligned} \tilde{\textbf{Y}} = \textbf{M} \! \odot \! \left( \mathcal {N} (\mathcal {S}_s (\mathcal {A}(\textbf{Y}))) \right) , \end{aligned}$$

where $\mathcal {A}: \mathbb {R}^{H \times W \times C} \rightarrow \mathbb {R}^{H \times W \times C}$ models optical blur, simulating the loss of high-frequency details due to lens aberration or motion; $\mathcal {S}_s: \mathbb {R}^{H \times W \times C} \rightarrow \mathbb {R}^{\frac{H}{s} \times \frac{W}{s} \times C}$ denotes downsampling by factor *s*, covering cases such as digital zoom-out, finite sensor resolution, or pipeline decimation; $\mathcal {N}: \mathbb {R}^{\frac{H}{s} \times \frac{W}{s} \times C} \rightarrow \mathbb {R}^{\frac{H}{s} \times \frac{W}{s} \times C}$ represents a noise corruption operator from sources like photon shot noise or sensor readout; and $\textbf{M} \in \{0,1\}^{\frac{H}{s} \times \frac{W}{s} \times C}$ is a binary mask modeling missing pixel degradation (e.g., scratches or random sensor failures). The element-wise multiplication ⊙ applies the mask to the observed signal. For additive noise, $\mathcal {N}(\textbf{Z})=\textbf{Z}+\boldsymbol{\eta }$, where $\boldsymbol{\eta }$ is the noise realization.

This unified formulation captures a wide range of real-world degradations—blur, noise, downsampling, and missing data. The goal of image restoration is to recover the clean image $\textbf{Y}$ from the observed input $\tilde{\textbf{Y}}$.

### Building a Strong Representational Foundation

We review implicit neural representations (INRs) for images, introduce a multi-grade extension of a plain (single-grade) INR, and discuss their benefits.

Rather than optimizing pixel values directly, an image can be represented by a neural network mapping 2D coordinates to pixel values. Let $\Omega =[-1,1]^2$ denote the normalized coordinate domain, $\textbf{x} \in \Omega$ a 2D coordinate, and *C* output channels (1 for gray scale, 3 for RGB). A *single-grade INR* defines a continuous function $f_{\theta }: \Omega \rightarrow \mathbb {R}^C$, where $f_{\theta }(\textbf{x})$ returns the pixel value at $\textbf{x}$. For a coordinate grid $\textbf{X} \in \Omega ^{H \times W}$, the latent image is obtained by evaluating the network at each coordinate:

$$\begin{aligned} \textbf{Y} = f_{\theta }(\textbf{X}), \quad \text {that is, }\ \textbf{Y}_{i,j} = f_{\theta }(\textbf{X}_{i,j}). \end{aligned}$$

This shifts the optimization from pixel space to network parameter space, leveraging the expressivity of neural networks to produce continuous, plausible image reconstructions.

#### Multi-Grade INR

*Definition of Single-Grade INR.* A *single-grade implicit neural representation* (SG-INR) is a network modeling a continuous mapping $f_\theta : \Omega \subset \mathbb {R}^2 \rightarrow \mathbb {R}^C$, where C denotes the number of channels. The output at $\textbf{x}$ is computed via a sequence of parameterized layers $\{\phi ^{(l)}_{\theta _l}\}_{l=1}^L$ and an output layer $\psi _{\theta '}$:

2 $$\begin{aligned} \begin{aligned}&\textbf{a}^{(0)} = \textbf{x}\in \Omega \subset \mathbb {R}^2,&\textbf{a}^{(1)} = \phi ^{(1)}_{\theta _1}(\textbf{a}^{(0)}), \\&\textbf{a}^{(l)} = \phi ^{(l)}_{\theta _l}(\textbf{a}^{(l-1)}),&f_\theta (\textbf{x}) = \psi _{\theta '}(\textbf{a}^{(L)}), \end{aligned} \end{aligned}$$

where $\phi ^{(1)}_{\theta _1}:\mathbb {R}^2\rightarrow \mathbb {R}^{d_\text {w}}$, $\phi ^{(l)}_{\theta _l}:\mathbb {R}^{d_\text {w}}\rightarrow \mathbb {R}^{d_\text {w}}$, l=2,⋯,L, and $\psi _{\theta '}:\mathbb {R}^{d_\text {w}}\rightarrow \mathbb {R}^C.$ Here $\textbf{a}^{(l-1)}$ and $\textbf{a}^{(l)}$ are the input and output feature vectors of the *l*th layer, respectively, and each $\phi ^{(l)}_{\theta _l}$ is a parameterized function (e.g., a fully connected layer with nonlinearity). The specific choice of $\phi ^{(l)}_{\theta _l}$ and $\psi _{\theta '}$ depends on the INR type (see Supp. Sect. A.1). All parameters $\theta =\{\theta _1,\dots ,\theta _L,\theta '\}$ are optimized jointly forming the baseline single-grade INR in our framework.

*Multi-Grade INR Structure and Training.* The multi-grade INR (MG-INR) is an additive stage-wise construction: for an INR with *L* hidden layers into *L* sequential *grades*. At grade *l* we append a new shallow network with one hidden layer $\phi ^{(l)}_{\theta _l}$ and one *dedicated* output layer $\psi _{\theta _l'}$ that learns the residual w.r.t the frozen aggregated model from previous grades. This converts the single large non-convex optimization into a sequence of smaller, tractable subproblems. Each grade *l* learns the **residual error** of the network up to grade l-1, and parameters from previous grades are frozen, acting as fixed adaptive “basis” functions [12].

Let $\textbf{x} \in \Omega \subset \mathbb {R}^2$ be an input coordinate. In grade *l* (l=1,⋯,L), the grade network $g^{(l)}$ computes the residual output:

3 $$\begin{aligned} \begin{aligned}&\textbf{a}^{(l)} {:}{=}\phi ^{(l)}_{\theta _l}(\textbf{a}^{(l-1)*}), \quad \text {with } \textbf{a}^{(0)*} = \textbf{x} \\&g^{(l)}_{\theta ^{(l)}}(\textbf{a}^{(l-1)*}) {:}{=}\psi _{\theta '_l}(\textbf{a}^{(l)}). \end{aligned} \end{aligned}$$

Here, $\textbf{a}^{(l-1)*}$ is the features learned in previous grades and serves as input in grade *l*, and $\theta ^{(l)} {:}{=}\{\theta _l, \theta '_l\}$ denotes the parameters specific to grade *l*.

The aggregated network function up to grade *l*, denoted $f^{(l)}$, is the sum of all individual grade outputs:

4 $$\begin{aligned} f^{(l)}_{\Theta _{\le l}^*}(\textbf{x}) = \sum _{j=1}^{l} g^{(j)}_{\theta ^{(j)*}}(\textbf{a}^{(j-1)*}). \end{aligned}$$

where $\Theta _{\le l}^* {:}{=}\{\theta ^{(1)*}, \dots , \theta ^{(l)*}\}$ represents the set of all optimal (frozen) parameters learned up to grade *l*.

*Training.* Training proceeds sequentially over grades l=1,⋯,L. At grade *l*, a new learnable function $g^{(l)}_{\theta ^{(l)}}(\textbf{a}^{(l-1)*})$ is added to the previously learned function, yielding the aggregated model

$$ f^{(l)}_{\theta ^{(l)}; \ \Theta _{\le l-1}^*}(\textbf{x}) = g^{(l)}_{\theta ^{(l)}}(\textbf{a}^{(l-1)*}) + f^{(l-1)}_{\Theta _{\le l-1}^*}(\textbf{x}), $$

where $\theta ^{(l)}$ in $g^{(l)}_{\theta ^{(l)}}$ are the only parameters to be trained in grade *l*. Grade *l* is trained by minimizing the discrepancy between the target $\textbf{y}$ (e.g., pixel value at $\textbf{x}$) and the aggregated output:

$$\begin{aligned} \theta ^{(l)*} = \textrm{argmin}_{\theta ^{(l)}} \mathcal {L}\left( f^{(l)}_{\theta ^{(l)}; \ \Theta _{\le l-1}^*}(\textbf{x}), \textbf{y}\right) . \end{aligned}$$

Here $\mathcal {L}$ denotes the training loss for the current grade, evaluated between the current multi-grade prediction $f^{(l)}_{\theta ^{(l)}; \ \Theta _{\le l-1}^*}(\textbf{x})$ and the image samples $\textbf{y}$. Crucially, during this step, the parameters from previous grades, $\Theta _{\le l-1}^*$, remain frozen, and only $\theta ^{(l)}$ is updated. The final MG-INR is the fully aggregated model, $f^{(L)}_{\Theta _{\le L}^*}(\textbf{x})$.

#### Advantages of Multi-Grade INR

In this subsection, we first present empirical comparisons between multi-grade and single-grade INR, showing that multi-grade INR consistently achieves superior reconstruction quality and representation capability. To understand the underlying reason for these improvements, we then investigate the learning dynamics of multi-grade INR and show that it effectively alleviates the spectral bias of single-grade INR. Finally, we discuss its optimization behavior and the resulting advantages.

**Fig. 2:**
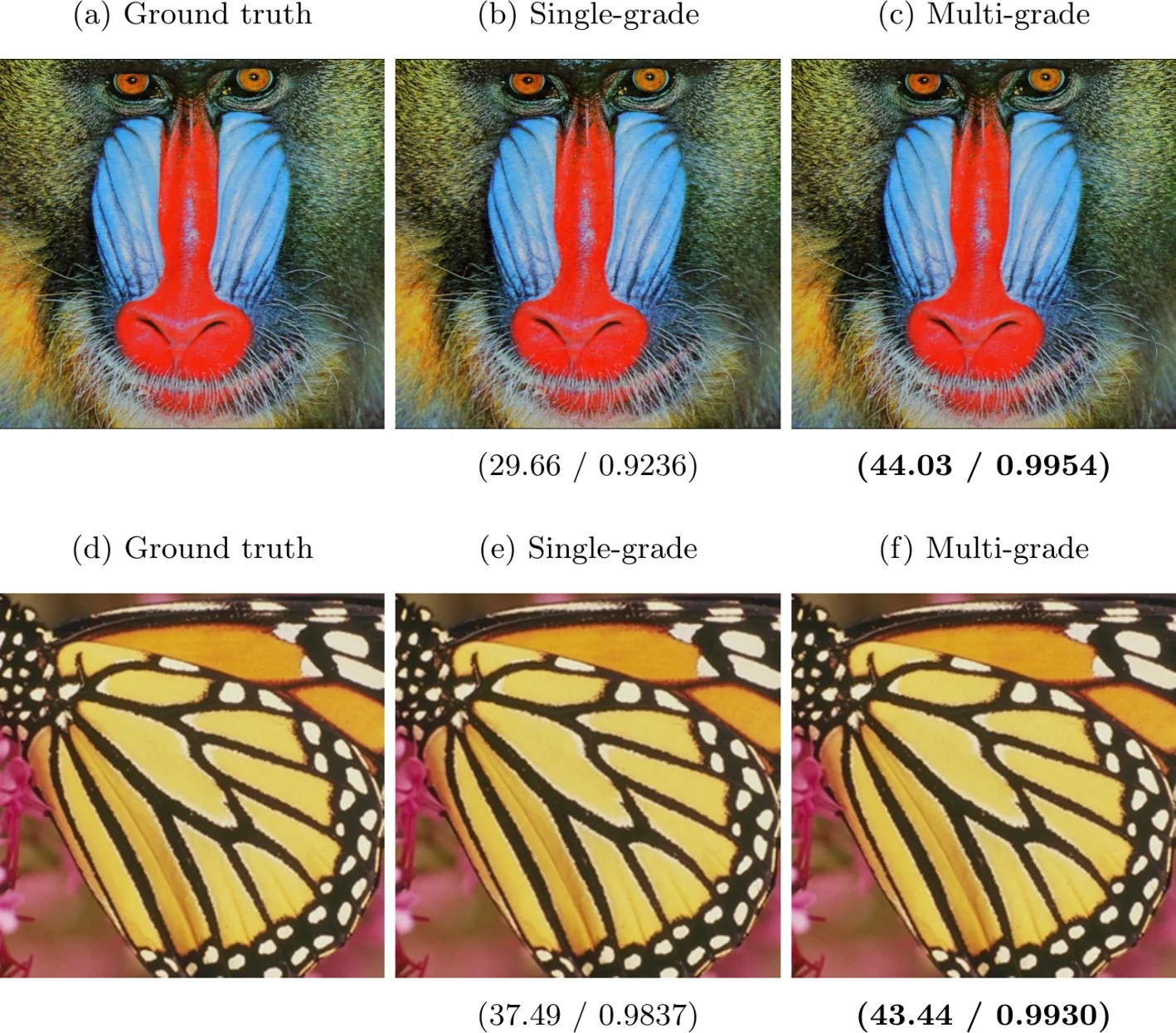
Multi-grade versus single-grade INR on a clean image-fitting task using SIREN. Because the representation capability of the INR backbone can upper-bound the achievable reconstruction quality in degraded cases, this fitting task evaluates whether the multi-grade design improves that capability

**Fig. 3:**
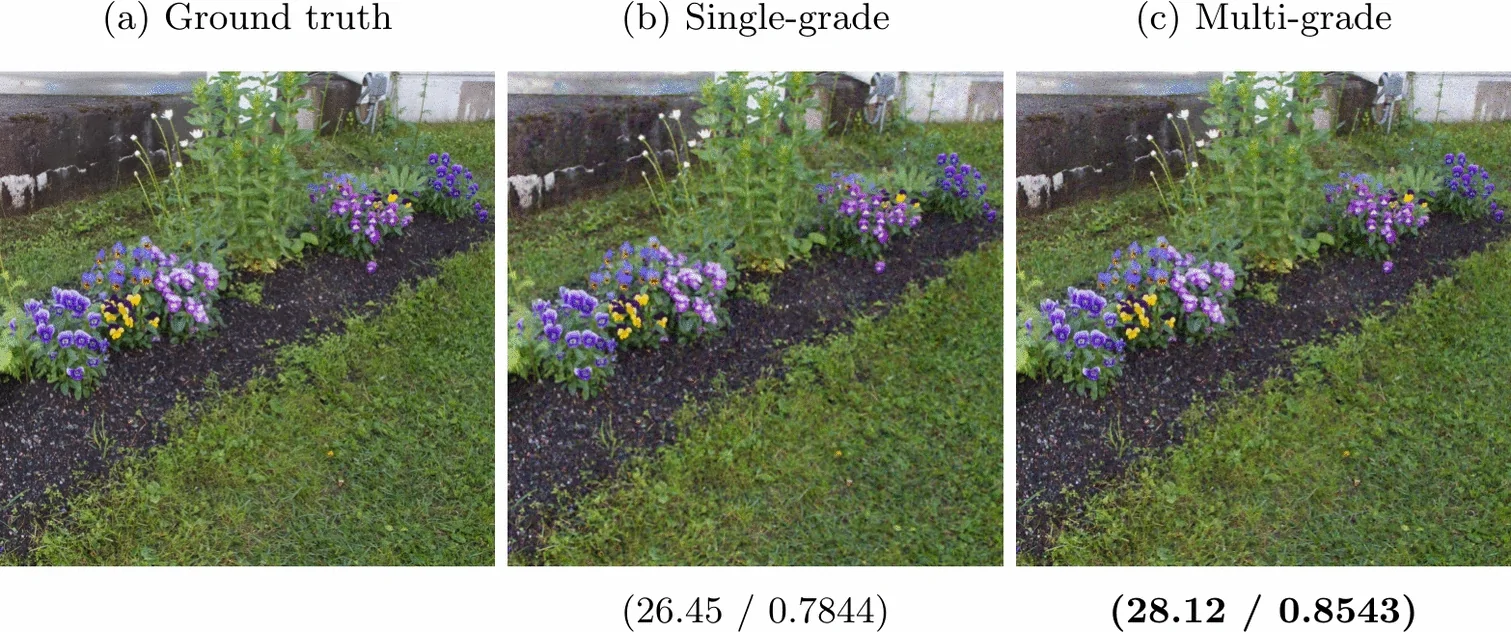
Multi-grade training improves denoising performance using SIREN with noise level $\sigma _\text {noise} = 15/255$

**Fig. 4:**
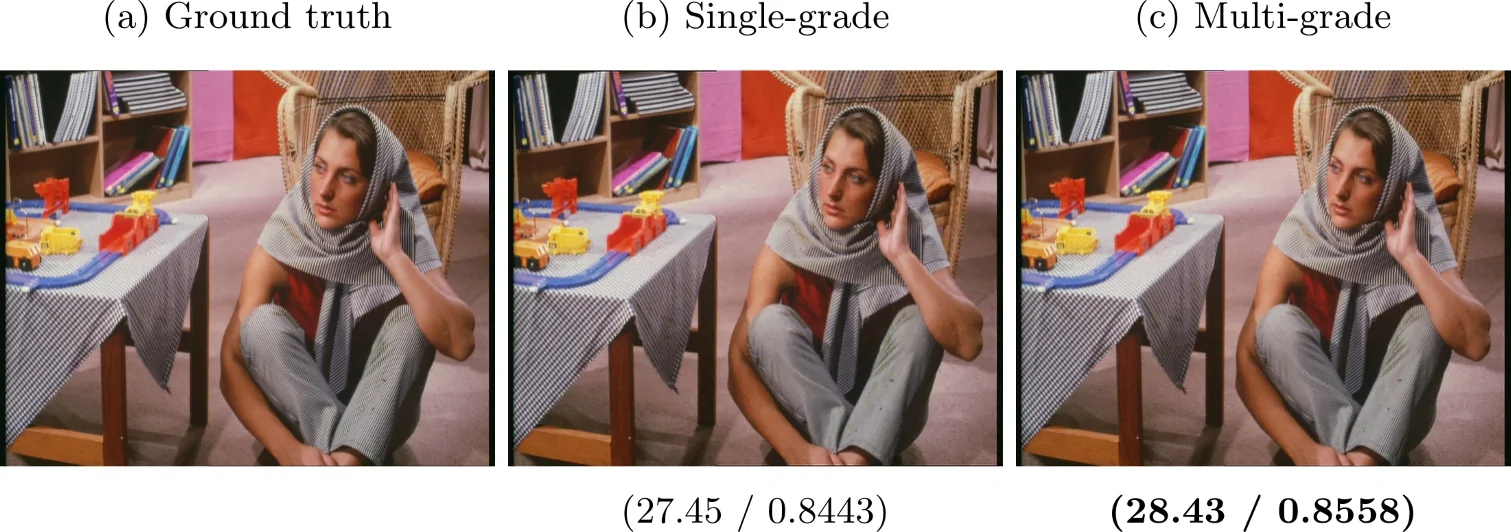
Multi-grade training improves deblurring performance using SIREN with $\sigma _\text {blur} = 1.0$ and $\sigma _\text {noise} = 3/255$

*Comparison of multi-grade and single-grade INR.* A comparison between multi-grade and single-grade INR on image fitting, denoising, and deblurring tasks is reported in Table 1, as well as in Fig. 2 (image fitting), Fig. 3 (image denoising), and Fig. 4 (image deblurring). Since MG-SpaIR reconstructs the unknown clean image by representing it with an INR, the INR backbone must first be able to represent clean image content accurately; otherwise, its limited representation capacity would become an upper bound on the reconstruction quality in degraded cases. Therefore, the clean image-fitting task in Fig. 2 is used as a degradation-free test of the basic representation capability of the multi-grade INR backbone. We observe that multi-grade INR outperforms single-grade INR in PSNR/SSIM by 14.37 dB/0.0718 on the Baboon image, and by 5.95 dB / 0.0093 on the Butterfly image. Figure 2 shows that multi-grade obtains sharper details than single-grade. These results indicate a clear improvement in representation capability.

On restoration tasks, multi-grade consistently outperforms single-grade, improving PSNR/SSIM by +1.67 dB/+0.0699 on denoising (Fig. 3) and +0.98 dB/+0.0115 on deblurring (Fig. 4). Moreover, it reduces VRAM usage by 30%. This balance of accuracy, efficiency, and quality makes the multi-grade strategy essential to our framework.

**Table 1:** Comparison of single-grade and multi-grade using SIREN on image fitting, denoising, and deblurring tasks

**Task**	**Image**	**Single-grade**		**Multi-grade**	
		**PSNR**	**SSIM**	**PSNR**	**SSIM**
Fitting	Baboon	29.66	0.9236	**44.03**	**0.9954**
	Butterfly	37.49	0.9837	**43.44**	**0.9930**
Denoising	Garden	26.45	0.7844	**28.12**	**0.8543**
Deblurring	Barbara	27.45	0.8443	**28.43**	**0.8558**

*Exploring the learning process of Multi-Grade INR.* To investigate how the multi-grade strategy improves representation, we revisit the Baboon image-fitting example in Fig. 2 and analyze the learned representation of each grade in the spectral domain. We visualize the logarithmic magnitude spectrum of the 2D Fourier transform of the incremental content contributed by each grade, together with the spectra of the ground truth and the final accumulated reconstruction $f^{(L)}$ (the grade-*L* output). After shifting the zero-frequency component to the center, low spatial frequencies appear near the Fourier origin, whereas higher spatial frequencies are located farther from the center.

**Fig. 5:**
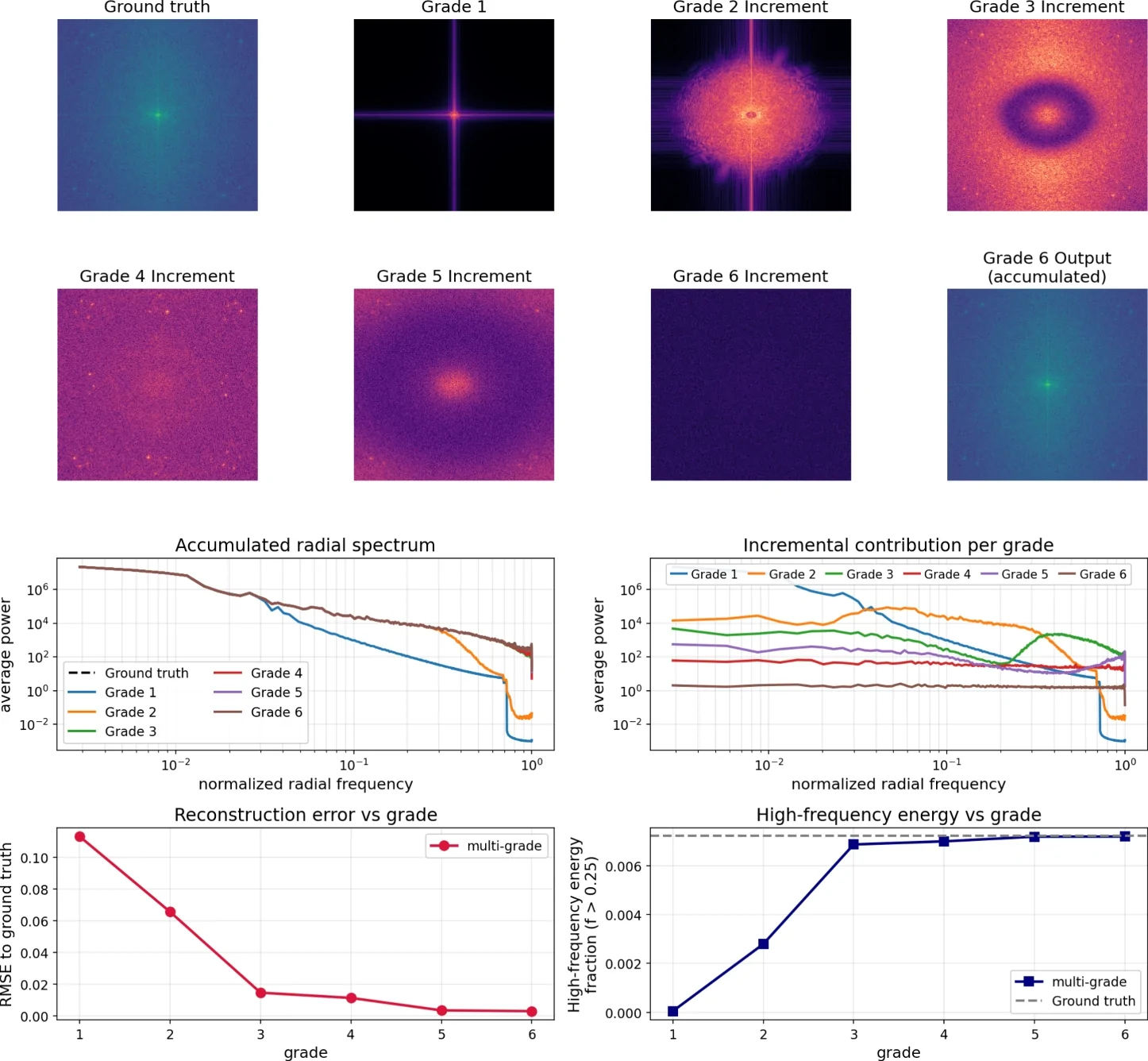
Per-grade spectral analysis of multi-grade INR on the Baboon image-fitting task. The top two rows contain the ground-truth log-magnitude Fourier spectrum, the Grade 1 spectrum (the first-grade output $g^{(1)}=f^{(1)}$), the Grade 2–6 incremental spectra (computed from the differences $g^{(l)}=f^{(l)}-f^{(l-1)}$), and the final Grade 6 accumulated spectrum $f^{(L)}$. The lower 2×2 block reports all-grade quantitative spectral domain curves: accumulated radial spectrum, incremental radial contribution per grade, RMSE to the ground truth, and high-frequency energy fraction

Overall, Fig. 5 shows quantitative trends consistent with progressive low-to-high-frequency refinement across grades: The accumulated radial spectrum approaches the ground truth, RMSE decreases and then saturates, and high-frequency energy rises toward the ground-truth level. At the stage level, earlier grades contribute the low-frequency components, whereas later grades add high-frequency details as the reconstruction approaches saturation. These observations are consistent with the role of the multi-grade strategy in alleviating the spectral bias observed in the single- versus multi-grade comparisons.

**Fig. 6:**
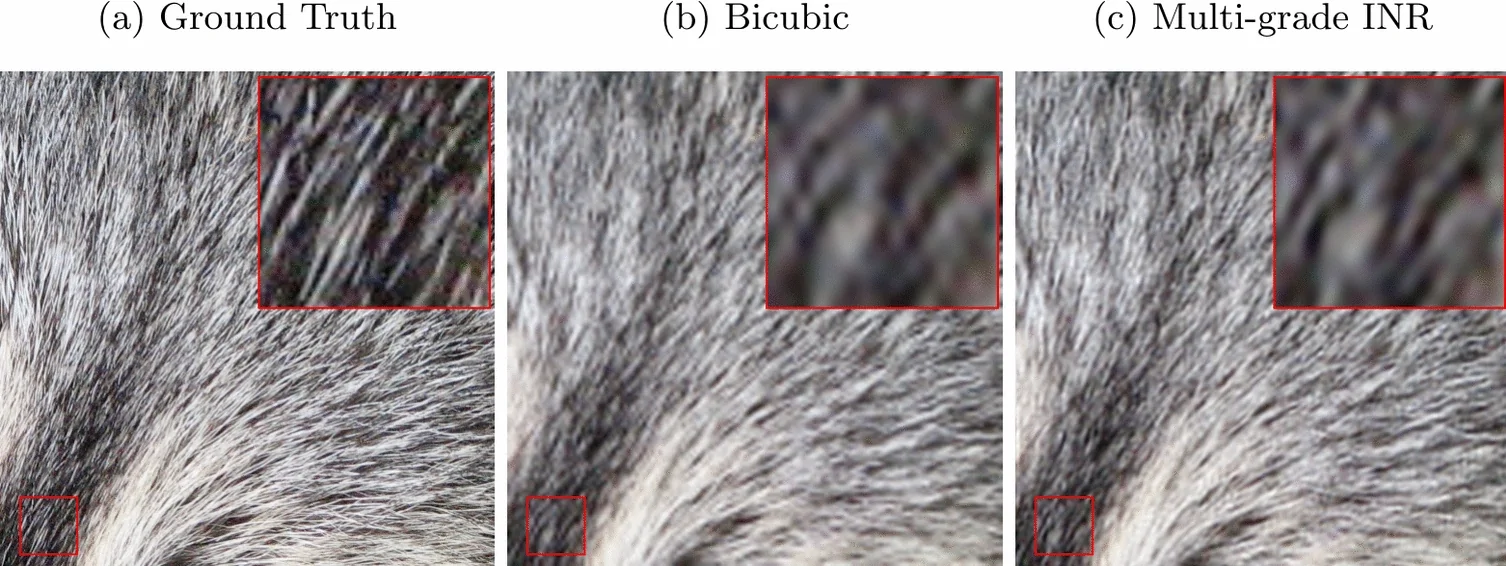
Comparison of the INR representation and bicubic interpolation on a super-resolution example

MG strategy offers several optimization advantages. By decomposing training into sequential grades, each solving a smaller and more tractable optimization problem, it alleviates gradient-related difficulties. With a single hidden layer and ReLU activation, each grade yields a convex optimization [38], making MG strategy effectively a sequence of convex subproblems [14]. Moreover, each grade can be analyzed independently, revealing how features accumulate and residuals diminish, which enhances interpretability. Computationally, MG strategy scales linearly with the number of grades and requires storing only the current grade’s parameters, unlike SG strategy, which updates all layers jointly. This multi-grade strategy increases INR expressivity, enabling the recovery of fine details from sparse or low-resolution inputs. As illustrated in Fig. 6, the INR-based reconstruction better preserves continuous structures than bicubic interpolation in this super-resolution example, highlighting the advantage of INR representations over interpolation-based reconstruction. Consequently, we adopt the multi-grade framework throughout this paper.

### Regularizing INRs

**Fig. 7:**
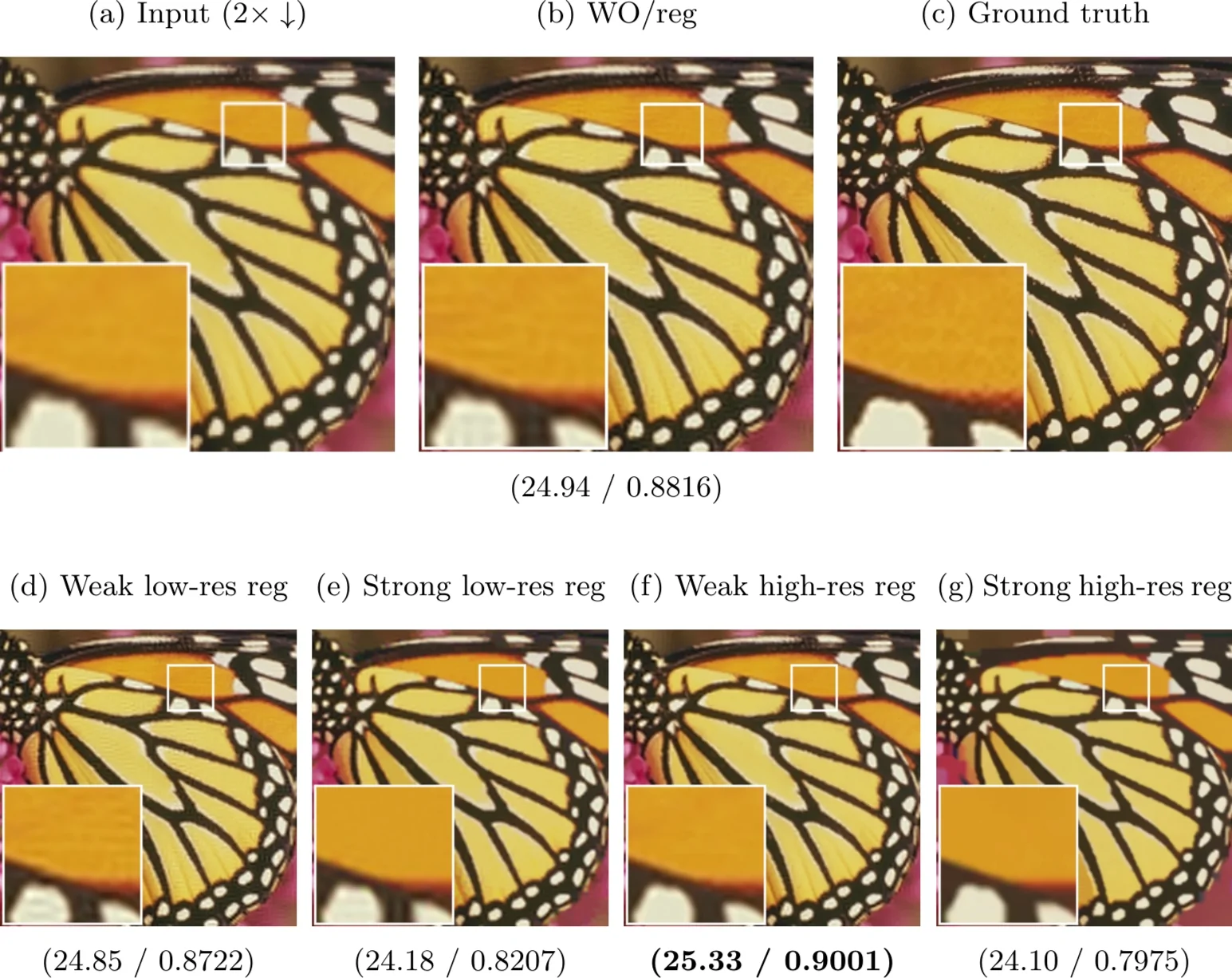
Comparison of regularization domains and strengths for super-resolution. Low-res/high-res specifies where the sparse proximal regularization is imposed, while weak/strong specifies the effective proximal regularization strength

**Fig. 8:**
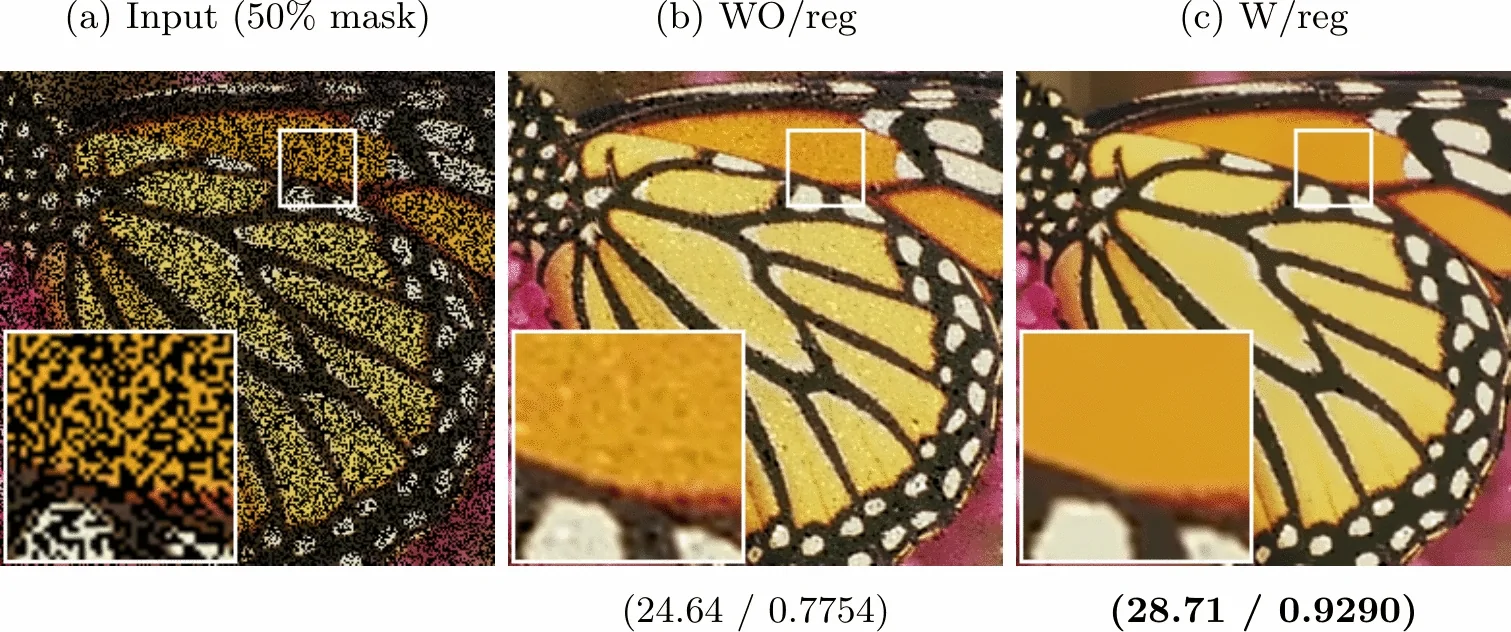
Necessity of regularization for inpainting

We further compare the role and placement of sparse proximal regularization in Figs. 7 and 8. The high-resolution regularization used by MG-SpaIR is formalized in Eq. (5). In Fig. 7, “high-res reg” applies the sparse proximal regularization to the gradient field of the high-resolution INR output, $f_\theta (\textbf{X}_\text {high})$, whereas “low-res reg” applies the corresponding regularization to the low-resolution INR output, $f_\theta (\textbf{X}_\text {low})$. Thus, low-res/high-res specifies the domain where the regularization is imposed; the data fidelity is still evaluated against the observed low-resolution image according to the degradation model. Independently, “weak” and “strong” indicate smaller and larger proximal regularization parameters, respectively.

As shown in Fig. 7, weak low-resolution regularization is insufficient to suppress artifacts, while strong low-resolution regularization and strong high-resolution regularization oversmooth the reconstruction. Weak high-resolution regularization achieves the best trade-off, suppressing artifacts while preserving fine details. Therefore, we use weak high-resolution regularization as the operating setting in MG-SpaIR. Figure 8 further shows that explicit regularization also substantially improves inpainting quality. This advantage of high-resolution regularization is further confirmed across INR backbones in the supplementary experiments.

### The MG-SpaIR Model

We now formalize our model. The objective is to learn INR parameters θ such that $f_{\theta }$, evaluated on a uniform grid, generates a clean high-resolution image whose forward-degraded version matches the observed input $\tilde{{\textbf{Y}}}$, while enforcing sparsity in its gradient field. Here $f_{\theta }(\textbf{X}_\text {high})$ denotes the INR $f_\theta$ evaluated on the high-resolution coordinate grid $\textbf{X}_\text {high}$ (specified below), and $\mathcal {S}_s$, $\mathcal {A}$, and $\textbf{M}$ are the downsampling, blur, and masking operators of the degradation model introduced in Sect. 3. These operators are assumed to be known in our formulation. To make this optimization tractable, we introduce an auxiliary variable $\textbf{U}$ representing the image gradients, leading to the following joint problem:

5 $$\begin{aligned} \min _{{\textbf{U}},\theta }&\underbrace{\mathcal {L}_{\text {fid}} (\tilde{{\textbf{Y}}} - {\textbf{M}} \!\odot \!( \mathcal {S}_s (\mathcal {A} (f_{\theta }(\textbf{X}_\text {high})))))}_{\text {Fidelity Term}}\nonumber \\ +&\underbrace{\frac{\beta }{2}\Vert {\textbf{U}}-\mathcal {B} (f_{\theta }(\textbf{X}_\text {high}))\Vert _{\text {F}}^2}_{\text {Surrogate Term}} \quad + \underbrace{\lambda \Phi ({\textbf{U}})}_{\text {Regularization Term}}. \end{aligned}$$

Here, $\tilde{{\textbf{Y}}}\in \mathbb {R}^{H_\text {low} \times W_\text {low} \times C}$ is the observed input, and $s\in \mathbb {N}_+$ denotes the super-resolution factor. The target resolution is $H_\text {high} = s H_\text {low}$ and $W_\text {high} = s W_\text {low}$, with the coordinate grid $\textbf{X}_\text {high}=\{\textbf{x}_{ij}^{\text {high}}\}_{i,j}\subset [-1,1]^2$ uniformly sampled into $H_\text {high}\times W_\text {high}$ points. Similarly, $\textbf{X}_\text {low}=\{\textbf{x}_{ij}^{\text {low}}\}_{i,j}\subset [-1,1]^2$ denotes the low-resolution coordinate grid. Throughout the paper, $\textbf{X}_\text {high}$ and $\textbf{X}_\text {low}$ are sets of grid coordinates rather than individual coordinate points, and $f_\theta (\textbf{X})$ denotes coordinate-wise evaluation over the whole grid. Equivalently, if $\textbf{X}=\{\textbf{x}_{ij}\}_{i,j}$ is stored as a coordinate grid, then $f_\theta (\textbf{X})$ denotes the stacked image whose (*i*, *j*)th pixel is $f_\theta (\textbf{x}_{ij})$. The arguments of $\mathcal {L}_{\text {fid}}$ are in the low-resolution observed domain, i.e., $\textbf{A} \in H_\text {low} \times W_\text {low} \times C$. The actual form of the loss $\mathcal {L}_{\text {fid}}$ depends on the assumed noise model. For additive Gaussian noise, we use the squared loss: $\mathcal {L}_{\text {fid}}(\textbf{A}){:}{=} \frac{1}{2} \sum _{h,w,c}\textbf{A}_{h,w,c}^2$, where $\textbf{A}$ has dimension $H_\text {low} \times W_\text {low} \times C$. Under additive white Gaussian noise, this squared loss data-fidelity term coincides (up to a constant) with the negative log-likelihood of the observation, so minimizing it corresponds to maximum-likelihood estimation [39, Chapter 3.1.1]

The second term in Eq. (5) introduces a quadratic penalty linking the auxiliary variable $\textbf{U}\in \mathbb {R}^{H_\text {high}\times W_\text {high}\times 2C}$ to the finite-difference gradient of the high-resolution INR output $f_\theta (\textbf{X}_\text {high})$. Here, $\Vert \cdot \Vert _{\text {F}}$ denotes the Frobenius norm (i.e., the square root of the sum of squared entries). This surrogate term serves two purposes: it provides a smooth relaxation of the sparsity prior, mitigating staircase artifacts, and it enables efficient optimization via a proximal update of the regularization term. In summary, $\textbf{U}$ is an auxiliary variable introduced by variable splitting: it decouples the non-smooth regularizer Φ from the data-fidelity and INR terms, so that the joint problem can be solved by alternating minimization—$\textbf{U}$ is updated in closed form through the proximity operator of Φ, while the network parameters θ are updated by gradient steps.

The operator $\mathcal {B}$ computes per-channel forward finite differences with Neumann (replicate) padding:

6 $$\begin{aligned} \mathcal {B} f_\theta (\textbf{X}_\text {high}) {:}{=} ( \mathcal {D}_x f_\theta (\textbf{X}_\text {high}),\, \mathcal {D}_y f_\theta (\textbf{X}_\text {high}) ), \end{aligned}$$

where $\mathcal {D}_x$ and $\mathcal {D}_y$ denote per-channel horizontal and vertical differences, respectively. Unlike the common isotropic formulation that merges these differences, we adopt an *anisotropic* stacking approach, which, as shown in Fig. 15, yields superior restoration quality—likely due to better preservation of directional information.

The final term, $\lambda \Phi (\textbf{U})$, imposes a sparse prior on the reconstructed image. Our formulation admits proximal-style updates of $\textbf{U}$, allowing the prior to be efficiently enforced whenever a closed-form update exists. The $\ell _0$ norm of $\textbf{U}$, denoted by $\Vert \textbf{U}\Vert _0$, counts the number of nonzero entries in $\textbf{U}$. In our experiments, we observe that $\ell _0$-norm regularization [15, 19, 40, 41] consistently outperforms alternative choices of Φ, such as $\ell _1$ [42–44] and MCP. Please refer to Fig. 16 for a comparison.

### MG-SpaIR Algorithm

Algorithm 1 efficiently solves the highly non-convex problem in Eq. (5) via a multi-grade strategy [12] with the proximal alternating minimization [45].

The algorithm solves Eq. (5) sequentially, starting from grade 1. In grade $l \ (l=1,\ldots ,L)$, the loss function is

7 $$\begin{aligned} \begin{aligned} \mathcal {L}_{l}(\textbf{U}, \theta ^{(l)})&{:}{=}\mathcal {L}_{\text {fid}} \big ( \tilde{{\textbf{Y}}} - {\textbf{M}} \!\odot \!( \mathcal {S}_s (\mathcal {A} (f^{(l)}_{\theta ^{(l)}; \Theta _{\le l-1}^*}(\textbf{X}_\text {high}))))\big )\\&+ \frac{\beta }{2}\Vert {\textbf{U}} - \mathcal {B} (f^{(l)}_{\theta ^{(l)}; \Theta _{\le l-1}^*}(\textbf{X}_\text {high}))\Vert _\textrm{F}^2 + \lambda \Phi ({\textbf{U}}). \end{aligned} \end{aligned}$$

The algorithm minimizes $\mathcal {L}_{l}(\textbf{U}, \theta ^{(l)})$ using a Gauss–Seidel-type block coordinate descent: at each epoch, ${\textbf{U}}$ is updated first using the current $\theta ^{(l)}$, followed by updating $\theta ^{(l)}$ with the new $\textbf{U}$. These two steps are repeated iteratively until convergence.

$\textbf{U}$**-step:** Here Φ denotes a sparsity-promoting function applied to the auxiliary gradient variable $\textbf{U}$. Through the quadratic penalty above, $\textbf{U}$ is linked to the finite-difference image gradient, so Φ encourages sparse image gradients. We consider $\ell _0$, $\ell _1$, and MCP (minimax concave penalty), which lead to different proximal thresholding operators.

In this step, $\textbf{U}$ is updated via a proximal operator:

8 $$\begin{aligned} \textbf{U} \leftarrow \operatorname {prox}_{\frac{\gamma \lambda }{\beta }\Phi }(\gamma \mathcal {B} (f^{(l)}_{\theta ^{(l)}; \Theta _{\le l-1}^*}(\textbf{X}_\text {high}))+(1-\gamma )\textbf{U}), \end{aligned}$$

to solve $\min _{\textbf{U}} \ \mathcal {L}_l(\textbf{U}, \theta ^{(l)})$ with $\theta ^{(l)}$ fixed. The proximal operator $\operatorname {prox}_{\frac{\gamma \lambda }{\beta }\Phi }(\cdot )$ has simple closed forms for these choices of Φ (Supp. Section A.2).

θ**-step:** With $\textbf{U}$ fixed, $\theta ^{(l)}$ is updated:

9 $$\begin{aligned} \theta ^{(l)} \leftarrow \operatorname {OptStep}\left( \theta ^{(l)}, \frac{\partial \mathcal {L}_l}{\partial \theta ^{(l)}}({\textbf{U}}, \theta ^{(l)})\right) , \end{aligned}$$

where $\operatorname {OptStep}(\cdot , \cdot )$ can be a single gradient descent or Adam step.

**Algorithm 1:**
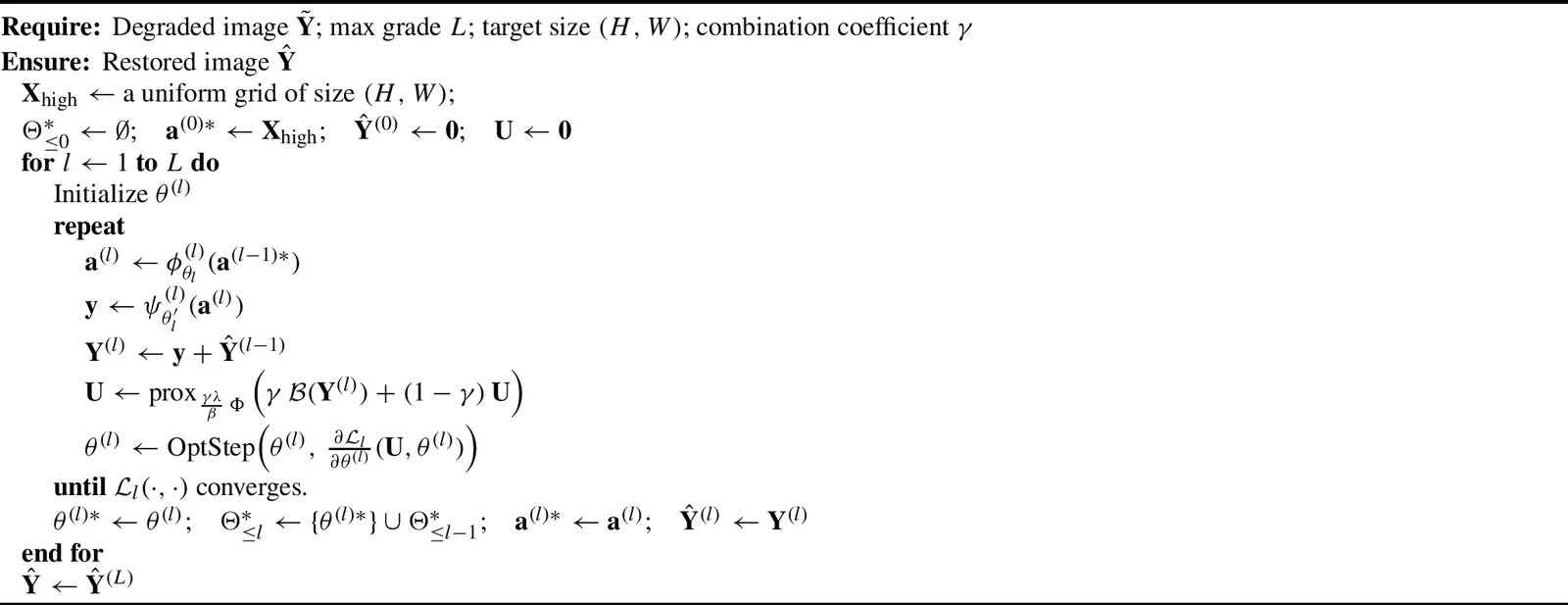
MG-SpaIR Algorithm

The computational cost of the MG-SpaIR Algorithm 1 mainly arises from updating $\textbf{U}$ and the network parameters θ. The update of $\textbf{U}$ involves evaluating the proximity operator $\textrm{prox}_{\frac{\gamma \lambda }{\beta }\Phi }$, which we assume admits a closed-form solution; several examples are provided in Appendix A.2. Therefore, the $\textbf{U}$-update is computationally inexpensive. The main computational burden lies in updating θ, i.e., training the neural network. Under the multi-grade strategy, a deep single-grade network is decomposed into a sequence of shallow networks, with each grade corresponding to one shallow network. If each grade has a comparable number of parameters and is trained for the same number of iterations, the total computational cost scales approximately linearly with the number of grades. Consequently, assuming the same number of iterations per grade as in the single-grade case, the overall computational cost of MG-SpaIR is comparable to that of a single-grade network with a similar total number of parameters.

## Convergence Analysis

We analyze the convergence of the MG-SpaIR algorithm.

For a fixed grade $l \in \{1, \ldots , L\}$, let $\mathcal {L}_\text {fid}$ denote the squared loss, Φ the $\ell _0$ regularization, and $\operatorname {OptStep}$ a gradient descent update with step size η. Define $f(\theta ^{(l)}) {:}{=}f^{(l)}_{\theta ^{(l)}; \Theta _{\le l-1}^*}(\textbf{x})$ and, for brevity, omit the superscripts of $\mathcal {L}_l$ and $\theta ^{(l)}$. Under these settings, the *k*th iteration of Algorithm 1 is

10 $$\begin{aligned} \begin{aligned}&\textbf{U}^{k+1} \in \operatorname {prox}_{\frac{\gamma \lambda }{\beta }\Phi }\!\bigl (\gamma \mathcal {B} (f(\theta ^{k})) + (1-\gamma )\textbf{U}^{k}\bigr ),\\&\theta ^{k+1} = \theta ^{k} - \eta \,\frac{\partial \mathcal {L}}{\partial \theta }(\textbf{U}^{k+1}, \theta ^k). \end{aligned} \end{aligned}$$

Here $\textbf{U}$ and θ (with or without superscripts) denote their flattened vectors of total dimension *d*.

If the activation of *f* is twice continuously differentiable, let $\boldsymbol{H}_\mathcal {L}(\textbf{U}, \theta )$ be the Hessian of $\mathcal {L}$ with respect to θ, and define

11 $$\begin{aligned} \alpha {:}{=} \sup \{\Vert \boldsymbol{H}_\mathcal {L}(\textbf{U}, \theta )\Vert _2 : (\textbf{U}, \theta ) \in \Omega \}, \end{aligned}$$

where $\Vert \cdot \Vert _2$ denotes the spectral norm.

### Theorem 4.1

Let $\{(\textbf{U}^k, \theta ^k)\}_{k=0}^\infty$ be generated by (10) with initial $(\textbf{U}^0, \theta ^0)$. Assume:

1. the activation function of the INR is twice continuously differentiable;
2. there exists a convex, compact $\Omega \subset \mathbb {R}^d$ such that
  $$ \{(\textbf{U}^k, \theta ^k)\}, \ \{(\textbf{U}^{k+1}, \theta ^k)\} \subset \Omega ; $$
3. α in (11) is finite.

If $\eta \in (0, 2/\alpha )$ and $\gamma \in (0,1)$, then:

(i) $$\displaystyle \lim _{k\rightarrow \infty }\mathcal {L}(\textbf{U}^k, \theta ^k) = L^*$$ for some $$L^* \ge 0$$;
(ii) $$\displaystyle \lim _{k\rightarrow \infty }\Vert \textbf{U}^{k+1} - \textbf{U}^k\Vert _2 = 0$$ and $$\displaystyle \lim _{k\rightarrow \infty }\Vert \theta ^{k+1} - \theta ^k\Vert _2 = 0$$;
(iii) $$\displaystyle \lim _{k\rightarrow \infty }\textrm{dist}\!\left( \boldsymbol{0}, \tfrac{\partial \mathcal {L}}{\partial \textbf{U}}(\textbf{U}^{k+1}, \theta ^k)\right) \!=\! 0$$ and $$\displaystyle \lim _{k\rightarrow \infty }\tfrac{\partial \mathcal {L}}{\partial \theta }(\textbf{U}^{k+1}, \theta ^k) = 0$$.

Here $\tfrac{\partial \mathcal {L}}{\partial \textbf{U}}$ denotes the subdifferential of $\mathcal {L}$ with respect to $\textbf{U}$, $\tfrac{\partial \mathcal {L}}{\partial \theta }$ its gradient with respect to θ, and $\textrm{dist}(\boldsymbol{p}, S){:}{=} \inf _{\boldsymbol{q}\in S}\Vert \boldsymbol{p}-\boldsymbol{q}\Vert _2$ is the distance from $\boldsymbol{p}$ to set *S*.

The proof of Theorem 4.1 is given in Appendix B. Theorem 4.1 establishes the convergence of Algorithm 1 under mild conditions when $\eta < 2 / \alpha$, where η is the learning rate and α the Hessian spectral norm. This result indicates that a smaller Hessian spectral norm permits a larger stable learning rate. Within each grade, the multi-grade strategy trains a shallow network with fewer parameters, resulting in a smaller Hessian spectral norm than its single-grade counterpart and thus allowing a higher learning rate. Moreover, the assumption that *f* is twice continuously differentiable is satisfied by the sinusoidal and Gabor-type activations used in our backbones (SIREN/WIRE), aligning the theory with our experimental setting.

**Fig. 9:**
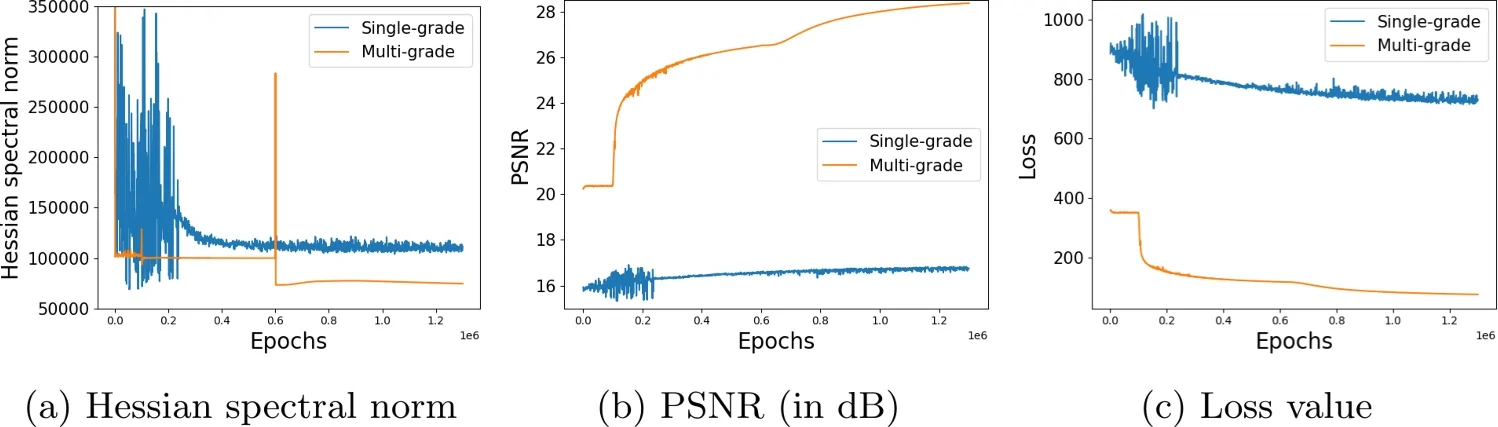
Evolution of Hessian spectral norm, PSNR, and loss during optimization for single-grade vs. multi-grade strategies ($\eta = 2\times 10^{-5}$)

To demonstrate this effect, we perform an image denoising experiment (Fig. 9). Both the single-grade and multi-grade networks, using a SIREN [4] backbone, are applied to the image denoising problem described in Sect. 3.3. The input image is corrupted by additive Gaussian noise with zero mean and a standard deviation of 10/255. For the single-grade network, four hidden layers are used. For the multi-grade network, three grades are employed: the first and second grades each contain one hidden layer, while the third grade contains two hidden layers. The learning rate is varied from $1\times 10^{-6}$ to $5\times 10^{-5}$. Both single-grade and multi-grade model are trained using Algorithm 1. The single-grade model achieves its highest PSNR at $\eta = 5\times 10^{-6}$, whereas the multi-grade model attains its peak PSNR at a larger learning rate, $\eta = 2\times 10^{-5}$. This observation indicates that the multi-grade model exhibits greater training stability under larger learning rates.

As shown in Fig. 9a, the multi-grade strategy consistently yields a smaller Hessian spectral norm, while Fig. 9b shows that, under the same learning rate, the single-grade strategy diverges, whereas the multi-grade one remains stable and continues to improve. These results confirm that the multi-grade strategy enables a larger stable learning rate.

Fig. 9c presents the loss value during optimization with a learning rate of $\eta = 2\times 10^{-5}$. The single-grade model exhibits pronounced oscillations throughout training and fails to achieve a low loss value. By contrast, the multi-grade model shows a smooth and stable decrease in loss, leading to better convergence. This behavior indicates that the multi-grade strategy improves the optimization landscape and enhances the stability and convergence of Algorithm 1.

## Experiments

We conduct experiments to evaluate MG-SpaIR by: (1) demonstrating its effectiveness on mixed-degradation tasks compared with neural network-based and traditional methods, (2) comparing with data-driven methods to illustrate the trade-off between reconstruction fidelity and hallucinated textures, and (3) validating key design choices through ablation studies. Additional ablations are provided in Supp.  Section C.

*Experimental Setup.* Center-cropped images from Set14 and Flickr2K are used as ground truths, degraded according to the mixed-degradation model in Eq. (1). The degradation combines several corruptions: $\mathcal {A}$, a Gaussian blur with kernel standard deviation $\sigma _\text {blur}=0.5$ or 1, and kernel size $2\lceil 3\sigma _\text {blur}\rceil + 1$; $\mathcal {S}_s$, a 2× bicubic downsampling; $\mathcal {N}$, additive Gaussian noise with $\sigma _{\text {noise}}=5/255$ or 10/255; and $\textbf{M}$, a random pixel mask with dropout probability $p_{\text {missing}}=0$, 0.1, or 0.2. The objective uses squared loss as $\mathcal {L}_{\text {fid}}$ and an $\ell _0$ norm as Φ. PSNR and SSIM values are reported below each image. The framework is implemented in PyTorch and executed on an RTX 4090 GPU with 24 GB of VRAM.

### Comparison with Regularized Methods

We evaluate MG-SpaIR on a challenging mixed-degradation task defined in Eq. (1), comparing it with two representative baselines: (1) a traditional restoration pipeline built from classical algorithms and (2) deep image prior (DIP) [3], which also exploits neural network structures for image restoration. To ensure fairness, DIP is optimized using a unified end-to-end loss rather than a sequential pipeline, which can accumulate errors.

The traditional baseline follows a sequential restoration process that inverts the degradation model. It consists of four stages: inpainting via the fast marching method [46], denoising with BM3D [2], bicubic super-resolution [47], and Wiener deblurring [48]. All degradation parameters are provided to their respective algorithms for a fair comparison. Further implementation details of this pipeline are given in the supplementary material.

**Fig. 10:**
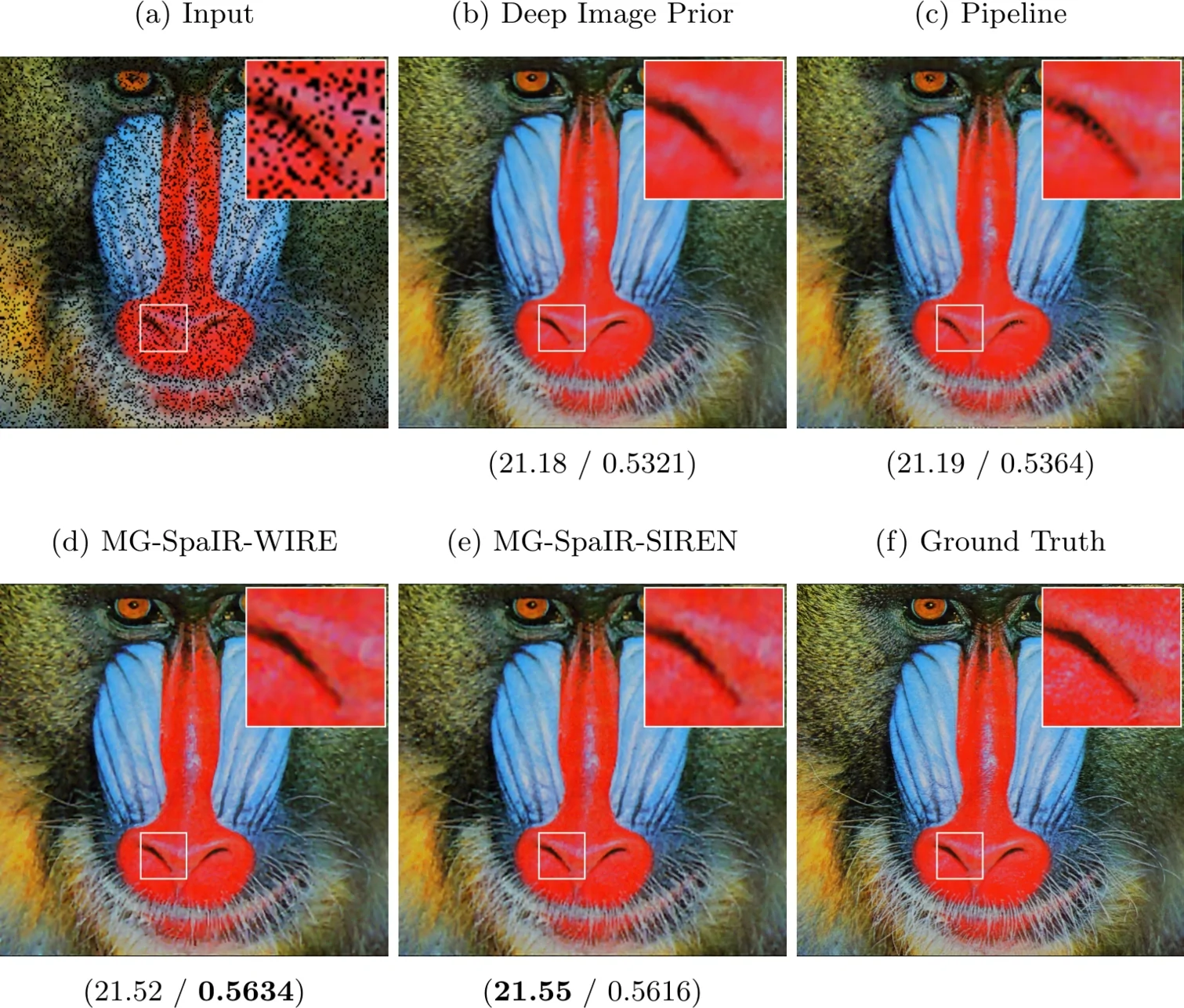
Image restoration of the Baboon image, showing finer detail recovery in irregular regions. Settings: $\sigma _\text {blur}=1$, 2× downsampling, $\sigma _\text {noise}=5/255$, $p_\text {missing}=0.2$

**Fig. 11:**
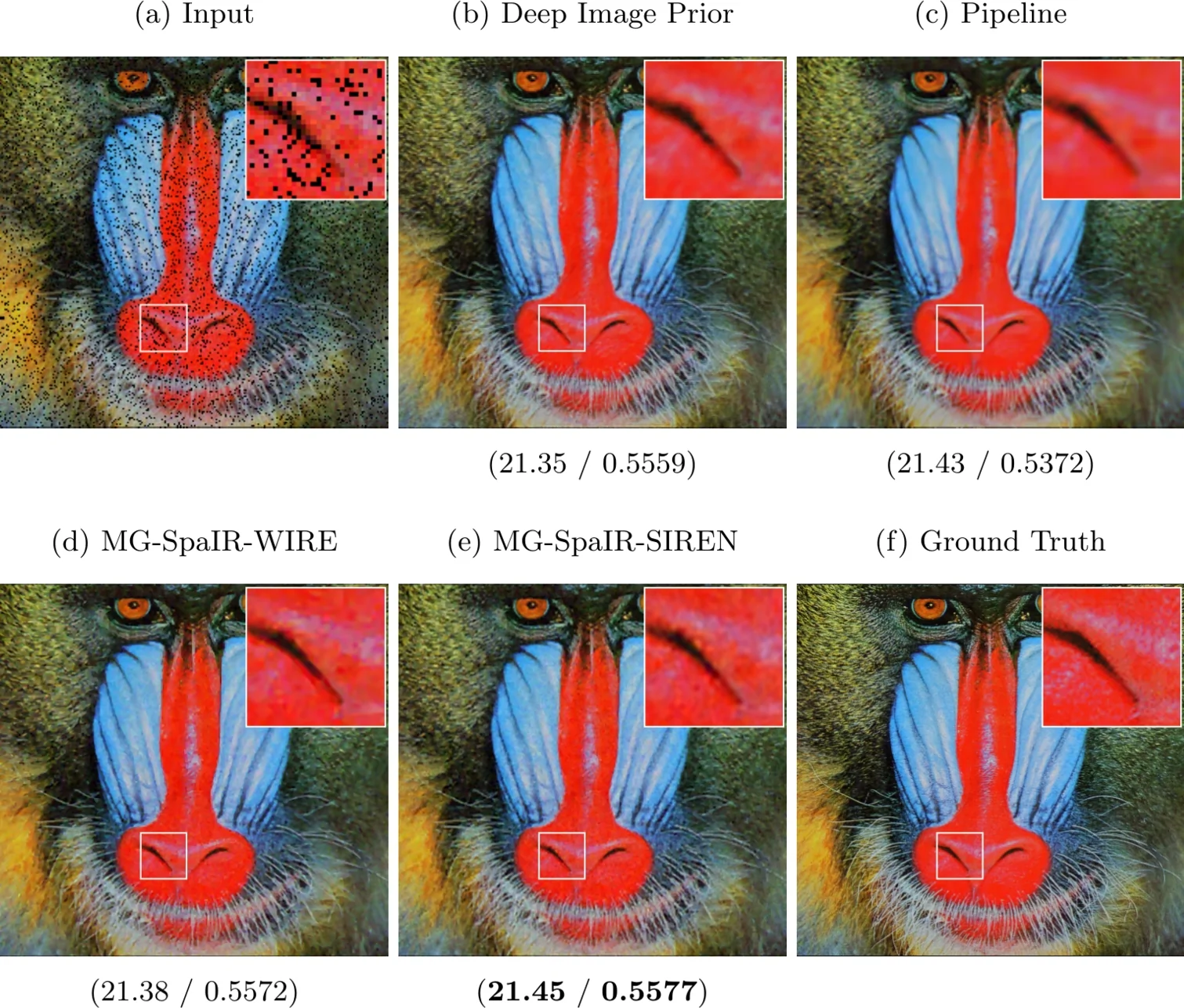
Image restoration of the Baboon image under another mixed-degradation setting. Settings: $\sigma _\text {blur}=0.5$, 2× downsampling, $\sigma _\text {noise}=10/255$, $p_\text {missing}=0.1$

**Fig. 12:**
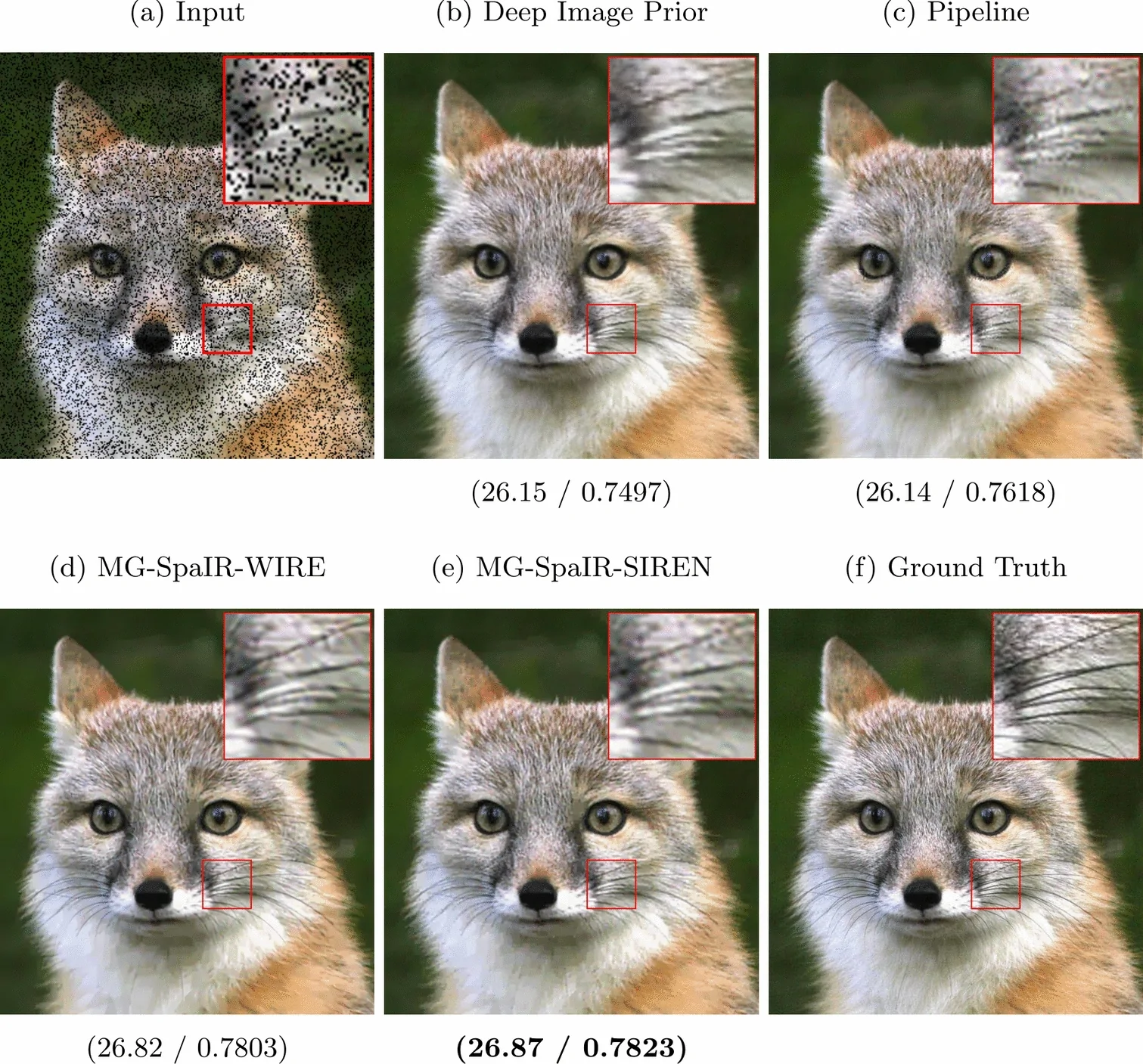
Image restoration of the Fox image, showing smoother and less pixelated line reconstruction. Settings: $\sigma _\text {blur}=1$, 2× downsampling, $\sigma _\text {noise}=5/255$, $p_\text {missing}=0.2$

**Fig. 13:**
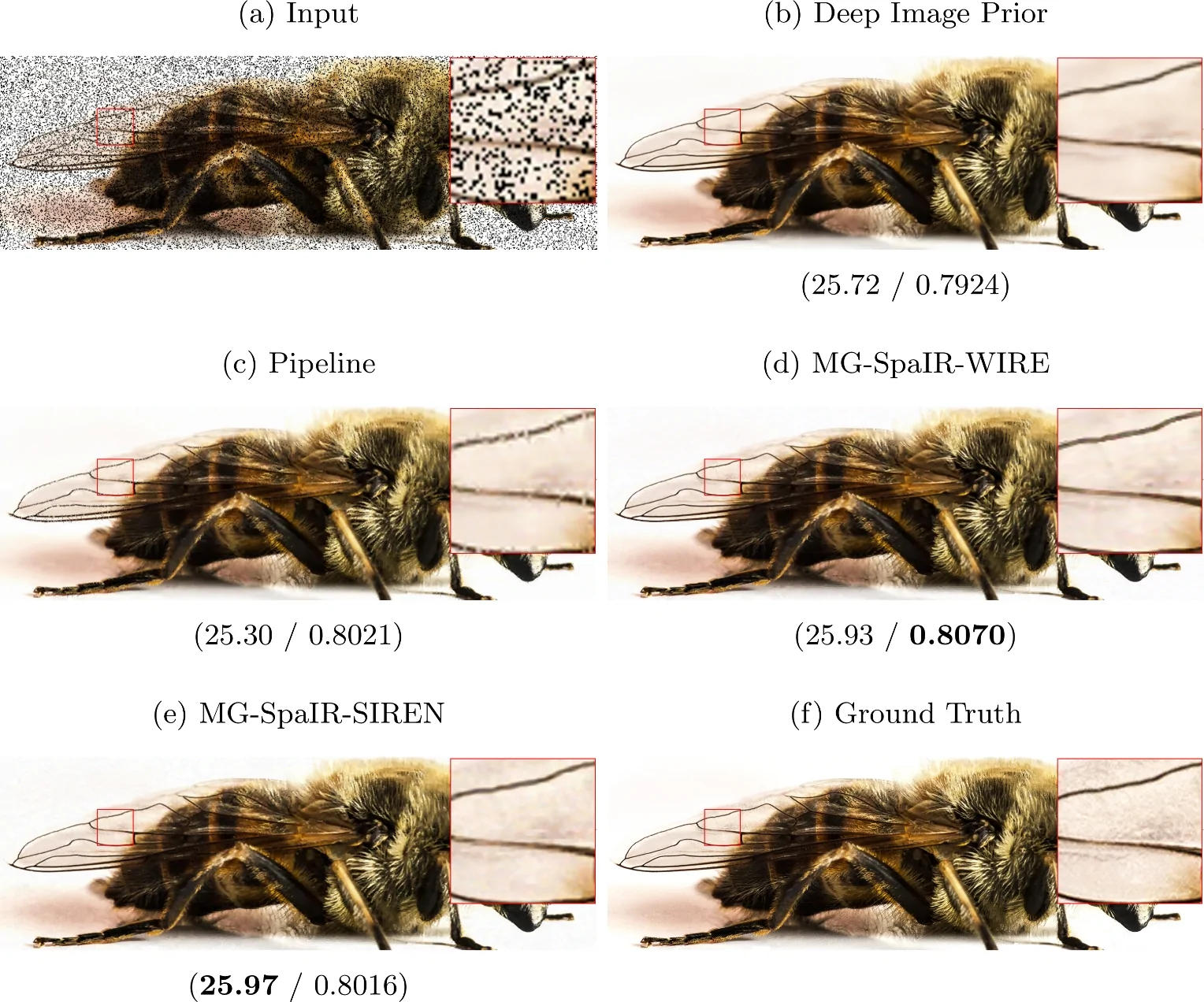
Image restoration of the Bee image, highlighting recovery of fine textures. Settings: $\sigma _\text {blur}=0.5$, 2× downsampling, $\sigma _\text {noise}=5/255$, $p_\text {missing}=0.2$

*Implementation.* MG-SpaIR can be instantiated with different implicit network backbones. Specifically, **MG-SpaIR-WIRE** corresponds to the case where $\phi _{\theta _l}^{(l)}$ and $\psi _{\theta _l'}$ follow Eqs. (A1) and (A2), respectively, while **MG-SpaIR-SIREN** uses the definitions in Eqs. (A3) and (A5), respectively. Both models are trained under the sparse proximal regularization framework introduced in Section 3.

Our methods, MG-SpaIR-WIRE and MG-SpaIR-SIREN, effectively reconstruct fine details (zoom-ins in Figs. 10 and 11) and smooth structures (zoom-ins in Figs. 12 and 13). DIP fails to recover irregular textures (Figs.10 and 11 and 13) and produces pixelated lines (Fig. 12). The traditional pipeline oversmooths fine details (Fig. 10) and fails to preserve structural lines (Fig. 13). The combined ability of our methods to preserve structure and recover detail results in the highest average PSNR and SSIM among all compared approaches.

### Comparison with Data-driven Methods

In this subsection, we compare our training-data-free multi-grade INR with a representative data-driven restoration method, SwinIR [22]. Unlike multi-grade INR, which is optimized directly from the observed input without external training data, SwinIR relies on restoration priors learned from large-scale training datasets and applies the learned model at inference time. The comparison is conducted under the same composite degradation setting, including blur, downsampling, and noise, but without missing pixels because SwinIR does not support inpainting. This experiment demonstrates that multi-grade INR achieves PSNR performance comparable to that of a data-driven transformer model, while requiring no external training data.

**Fig. 14:**
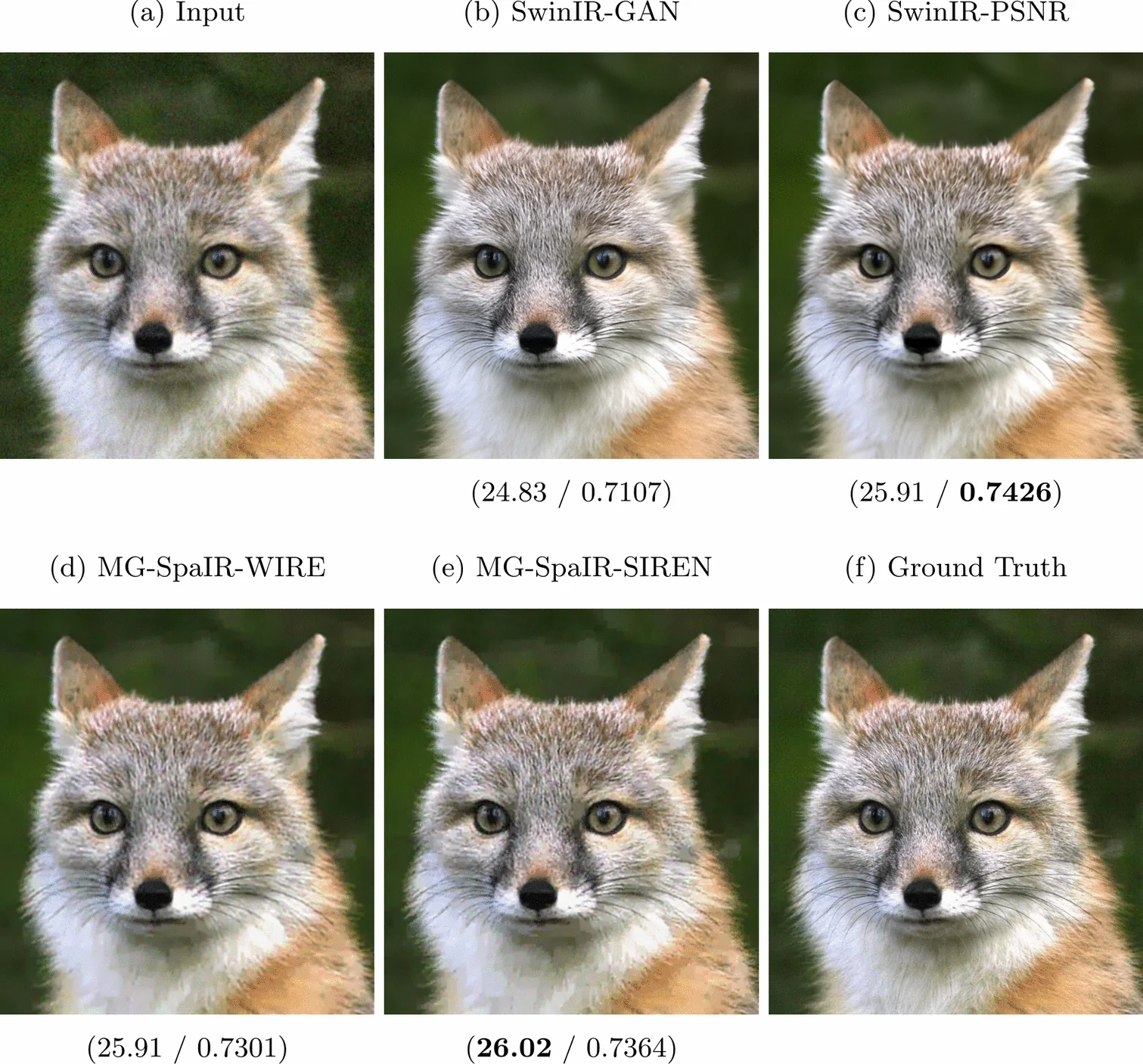
$\sigma _\text {blur}=1$, 2× downsampled, $\sigma _\text {noise}=10/255$, $p_\text {missing}=0$ (No missing pixels)

The detailed implementation and inference settings for this baseline are provided in Appendix C.1. This comparison is not intended to claim that MG-SpaIR generally outperforms data-driven learning methods. Our MG-SpaIR-based results (Fig. 14d and e) achieve comparable PSNR to the SwinIR baselines, while SwinIR-PSNR still obtains the higher SSIM. More broadly, Fig. 14 illustrates a distortion–perception trade-off, where the learning-based SwinIR results are visually cleaner and sharper, though MG-SpaIR preserves mild high-frequency noise and appears less smooth .

Figure 14 presents a qualitative comparison on a representative test example. The results should be interpreted as a fidelity/perception trade-off rather than as a claim that our training-data-free method surpasses learning-based restoration methods in general. Our MG-SpaIR-based results (Fig. 14d and e) achieve comparable PSNR to the SwinIR baselines; however, SwinIR-PSNR obtains the highest SSIM, and the SwinIR variants (Fig. 14b and c) are visually cleaner and sharper. MG-SpaIR preserves mild high-frequency noise and appears less smooth, although it remains competitive on distortion metrics in this example.

Unlike data-driven models such as SwinIR, which may hallucinate textures due to reliance on external training data, our method is guided solely by implicit priors from the INR backbone and explicit handcrafted sparsity priors applied in the image domain. These two complementary priors jointly steer the optimization toward faithful structures rather than invented details.

### Ablation Studies

**Fig. 15:**
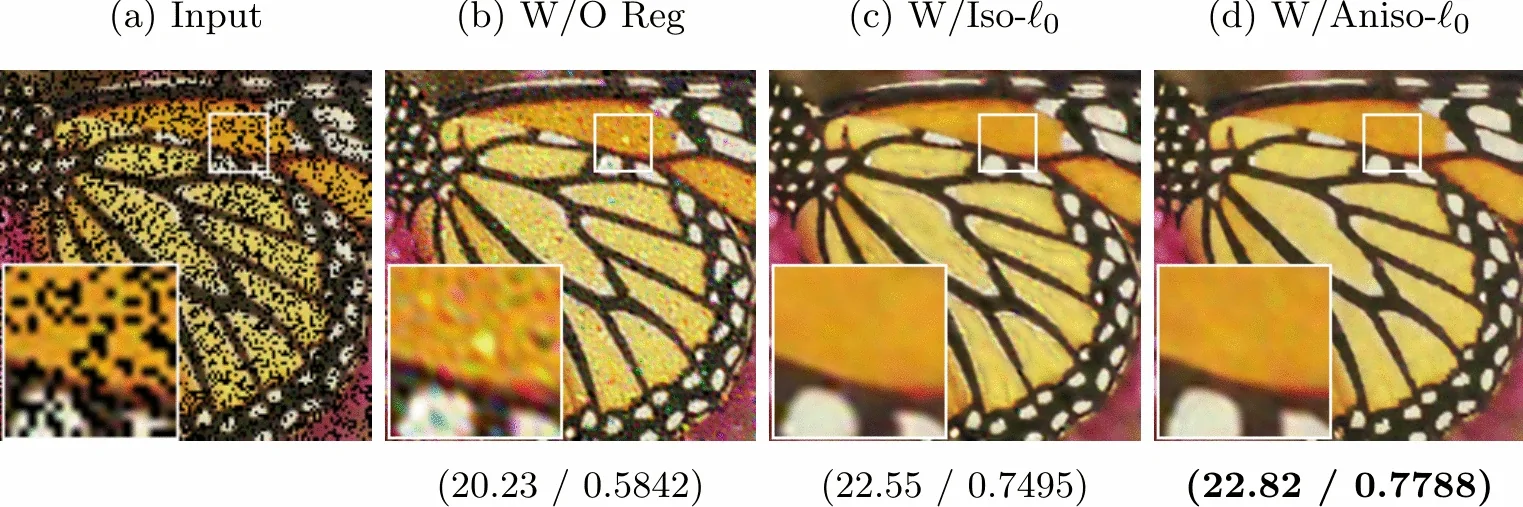
Ablation study on the regularization prior. From left to right: observed input, reconstruction without an explicit prior, with isotropic $\ell _0$ regularization, and with our proposed anisotropic $\ell _0$ regularization (Ours). Results demonstrate the need for an explicit prior and the superiority of the anisotropic formulation

*Necessity of Anisotropic* $\ell _0$. To evaluate our key design choices, we perform an ablation study on the regularization prior. The visual and quantitative results in Fig. 15 support two main conclusions.

First, the results confirm the necessity of an explicit prior. As shown in Fig. 15b, relying solely on the INR’s implicit regularization is insufficient, producing reconstructions with significant residual noise and low PSNR. The corresponding computational cost is discussed in Sect. 5.5.1.

Second, the formulation of the $\ell _0$ prior is critical. Figure 15d demonstrates that the anisotropic version achieves superior restoration quality with the highest PSNR and SSIM, thanks to its ability to preserve directional information. In contrast, the isotropic $\ell _0$ norm (Fig. 15c), applied to the gradient magnitude by combining horizontal and vertical derivatives, can lose orientation cues that guide INR optimization. Our anisotropic formulation decouples these components, allowing independent regularization and improved reconstruction.

### Effectiveness across Different Sparsity Regularizers

**Fig. 16:**
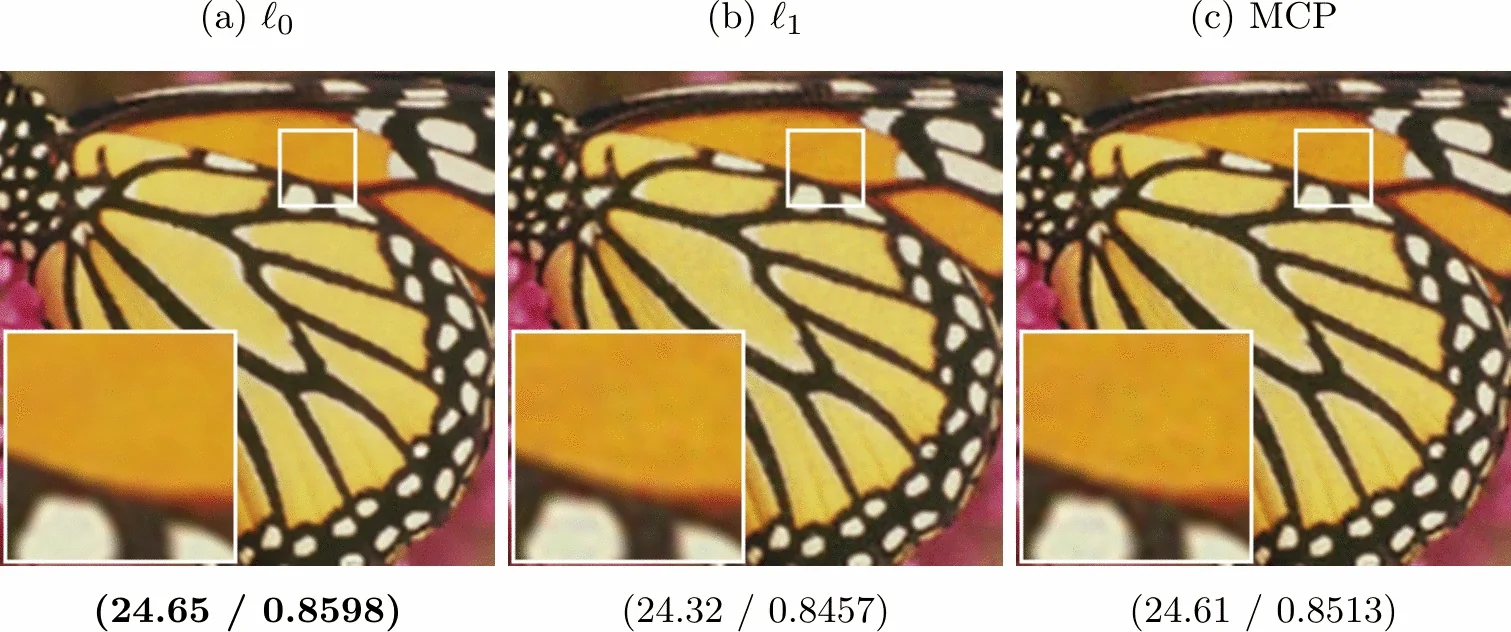
Effectiveness of our method with different sparse regularizations

To demonstrate the versatility of our proposed method, we apply it to several distinct sparsity regularization including $\ell _0, \ell _1$, and MCP. As shown in Fig. 16, our method performs consistently across different sparsity regularizers, with $\ell _0$ achieving the best performance.

### Effectiveness across Different INR Backbones.

**Fig. 17:**
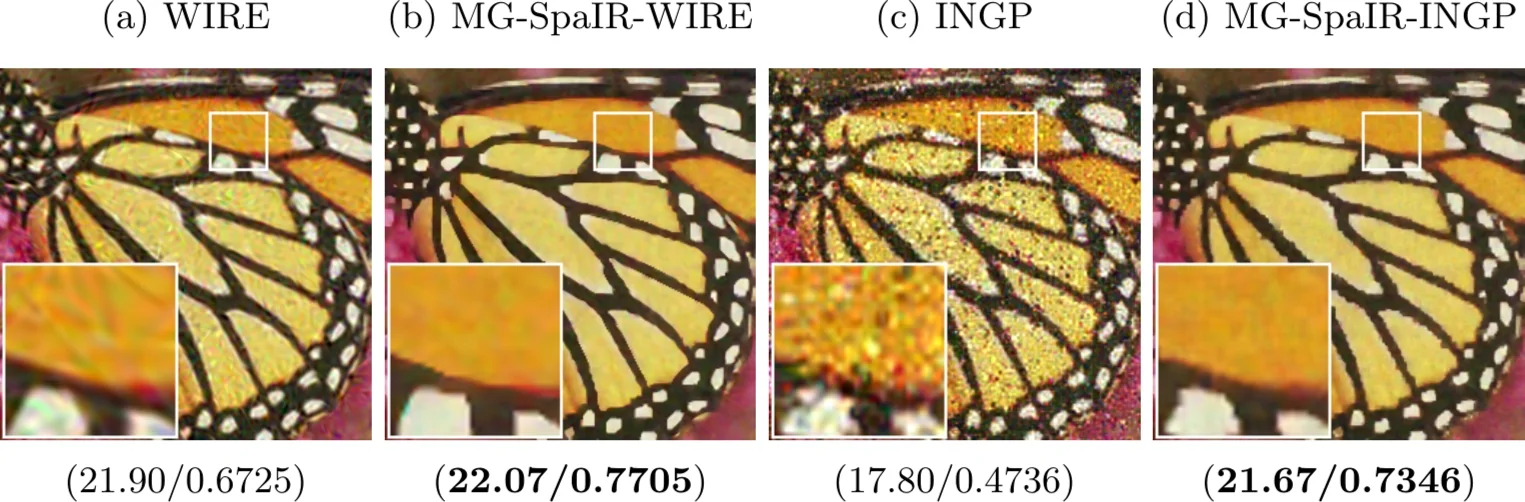
Effectiveness of our regularization across different INR backbones. Values are PSNR/SSIM

To demonstrate the versatility of our proposed regularization, we apply it to several distinct INR backbones. As shown in Fig. 17, our method consistently improves the performance of MLP-based backbones such as SIREN, Gabor-based backbones such as WIRE, and hash-grid-based backbones such as Instant-NGP (“INGP”) [5]. This confirms that our proposed prior is a model-agnostic module beneficial for a wide range of INR frameworks. Although the INR itself has implicit regularization [6], this experiment shows that adding an explicit regularization for image restoration is helpful and necessary. The corresponding computational cost is discussed in Sect. 5.5.1.

**Fig. 18:**
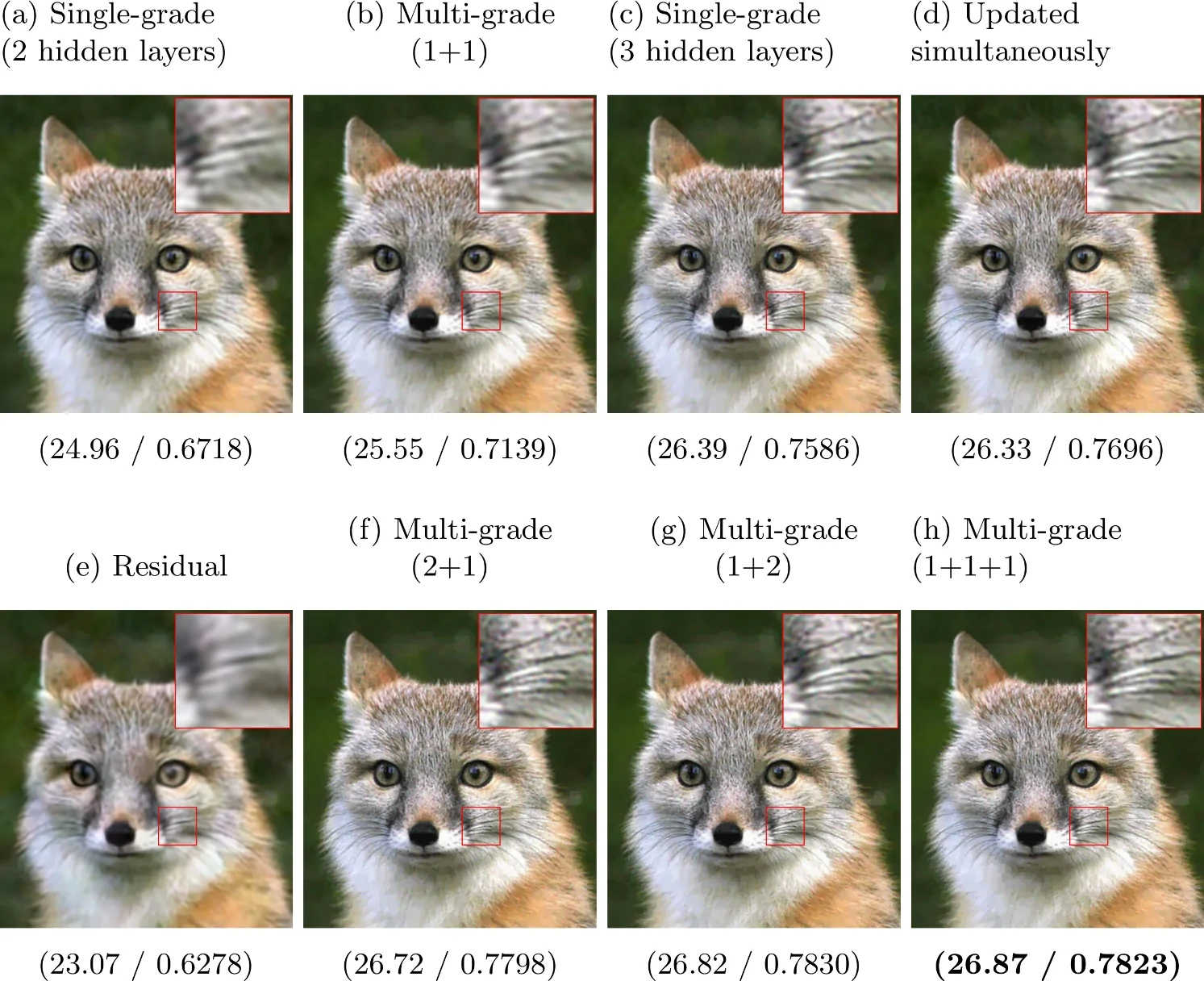
Effectiveness of the proposed multi-grade strategy. Single-grade entries are labeled by their total number of hidden layers, while multi-grade (a+b+⋯) denotes consecutive grades with a,b,… hidden layers. Values are PSNR/SSIM

*Effectiveness of the Multi-Grade Strategy.* To evaluate the effectiveness of the multi-grade strategy, we compare it using SIREN against three counterparts: a plain single-grade SIREN, an architectural variant with residual connections (“Residual”) [49], and a variant (“Updated simultaneously”) that uses the same multi-grade architecture and updates all grade parameters simultaneously from the beginning . The visual results in Fig. 18 show that the multi-grade strategy achieves higher PSNR and SSIM than both the single-grade approach and the Updated simultaneously counterpart. Notably, the residual connection variant suffers from oversmoothing and loss of fine textures, resulting in worse scores than the plain single-grade model.

The comparisons in Fig. 18 address two separate questions. First, to study the effect of the number of grades while keeping the overall network depth fixed, we compare depth-3 models with one grade (single-grade, Fig. 18c), two grades (2+1 and 1+2, Fig. 18f and g), and three grades (1+1+1, Fig. 18h), where a+b+⋯ denotes consecutive grades with a,b,… hidden layers, respectively. The models with two or three grades outperform the single-grade baseline, showing the benefit of using multiple grades at matched total depth. Second, to study the effect of network depth, we compare matched depth pairs: depth 2 single-grade (Fig. 18a) versus multi-grade (1+1) (Fig. 18b), and depth 3 single-grade (Fig. 18c) versus multi-grade (1+1+1) (Fig. 18h). In both depth settings, the multi-grade methods achieve higher PSNR and SSIM than the corresponding plain FNNs (single-grade), indicating that the improvement is consistent under different network depths.

In particular, simply updating all grades simultaneously from initialization does not recover the benefit of the proposed stage-wise multi-grade optimization. Updated simultaneously obtains 26.33 dB / 0.7696 SSIM, whereas multi-grade obtains 26.87 dB / 0.7823 SSIM.

#### Computational Cost

Tables 2, 3, and 4 summarize the training loop runtime for three comparisons: the comparison with and without sparse proximal regularization using the SIREN backbone, the comparison with and without sparse proximal regularization across different INR backbones in Fig. 17, and the multi-grade strategy. AMP denotes PyTorch automatic mixed precision, which uses mixed-precision computation to accelerate training. For multi-grade, enabling AMP reduces PSNR/SSIM only slightly, from 26.87/0.7823 to 26.79/0.7839. For the multi-grade comparison, single-grade, Updated simultaneously, and multi-grade use the same depth and width, with nearly identical total trainable parameter counts (528,387 for single-grade and 531,465 for Updated simultaneously and multi-grade). The single-grade and Updated simultaneously variants use 7000 optimizer steps, while multi-grade uses 7000 steps per grade.

**Table 2:** Training loop runtime with and without regularization

**Variant**	**Without regularization**	**With regularization**
**Time (s)**	119.7	164.8

**Table 3:** Runtime for the with/without regularization comparison across INR backbones in Fig. 17

**Variant**	**WIRE**	**MG-SpaIR-WIRE**	**INGP**	**MG-SpaIR-INGP**
**Time (s)**	28.1	66.9	7.66	7.74

**Table 4:** Training loop runtime for the multi-grade strategy

**Variant**	**Time without AMP (s)**	**Time with AMP (s)**
Single-grade	380.5	218.6
Updated simultaneously	487.0	273.9
Multi-grade	401.8	247.3

## Conclusion

We introduced MG-SpaIR, a training-data-free image restoration framework that unifies denoising, deblurring, super-resolution, and inpainting within a single self-supervised optimization model. MG-SpaIR combines a multi-grade implicit representation—trained sequentially in a progressive, residual manner that adds finer high-frequency detail across grades—with a sparse proximal regularization applied in the high-resolution image domain to stabilize fitting and suppress INR-induced artifacts. The multi-grade construction improves representational fidelity and optimization stability by decomposing training into a sequence of smaller subproblems, enabling sharper reconstructions and reduced sensitivity to learning rate choices. The sparse proximal prior further steers the optimization toward clean, structurally faithful solutions, reducing spurious high-frequency artifacts and residual noise. Experiments on mixed-degradation settings demonstrate that MG-SpaIR achieves state-of-the-art performance among training-data-free INR-based methods and offers a stable, interpretable alternative to data-driven restoration.

## Figures and Tables

**Fig. 19:**
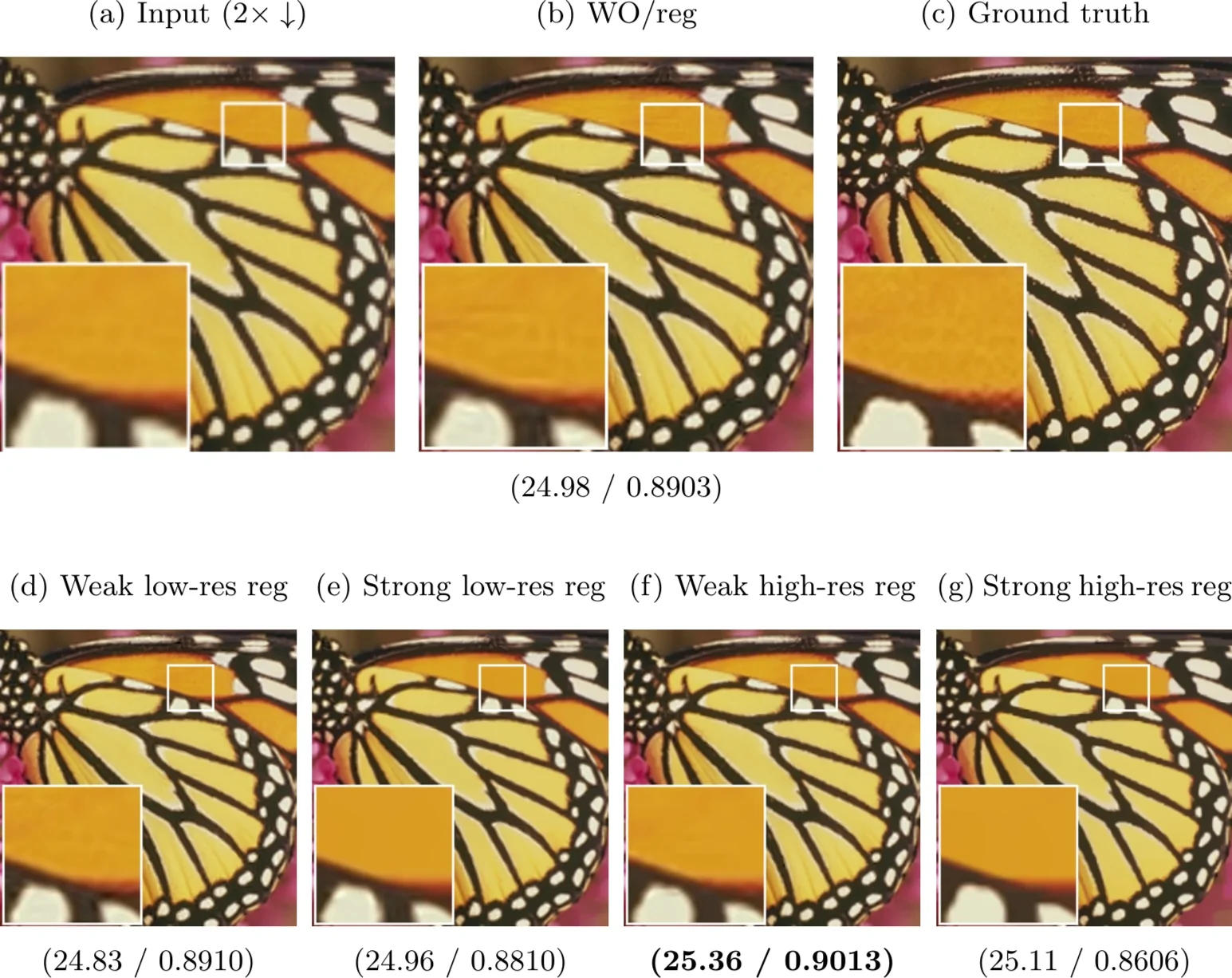
Comparison of results using different regularizations for the super-resolution task with WIRE.

**Fig. 20:**
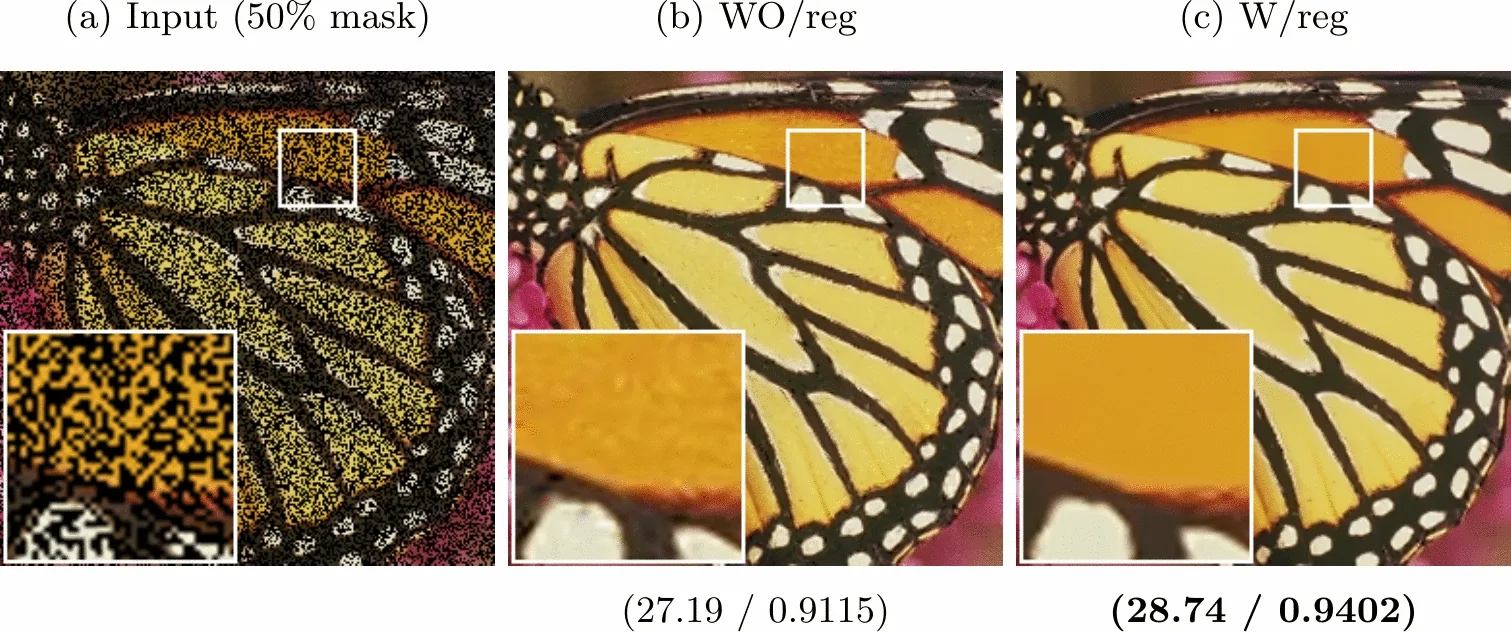
Comparison of inpainting results with and without regularization using WIRE.

## Data Availability

The datasets analyzed during the current study are publicly available. The Set5 and Set14 datasets are standard benchmarks in the field and are widely available in public repositories (e.g., https://www.kaggle.com/datasets/ll01dm/set-5-14-super-resolution-dataset). The Flickr2K dataset was introduced by Lim et al. and is available for download in public repositories (e.g., https://www.kaggle.com/datasets/daehoyang/flickr2k). Additionally, the specific image “The quick brown fox” used in this study is available on Wikimedia Commons (https://commons.wikimedia.org/wiki/File:The_quick_brown_fox....._(15677707699).jpg).

## Implementation Details: INR Backbones and Proximal Operators

This section presents the implementation details, including the INR backbones and the closed-form proximal operators required by our optimization algorithm.

### Layer Functions in INR Backbones

The proposed framework can be directly applied to various foundational INR backbones [5, 7, 11] for image restoration. In particular, we adopt two representative backbones, WIRE [6] and SIREN [4], as illustrative backbones in our experiments. WIRE introduces localized frequency modulation inspired by wavelets, while SIREN captures globally coherent high-frequency details through sinusoidal activations. Both backbones have been explicitly evaluated on image generalization tasks in their original works and achieved state-of-the-art performance, making them ideal backbones for our framework.

#### WIRE.

The WIRE architecture [6] employs a layer function based on the continuous complex 2D Gabor wavelet. For the WIRE network, each hidden layer is defined as

A1 $$\begin{aligned} \begin{aligned} \phi ^{(l)}_{\theta _l}(\textbf{a}^{(l-1)}) = \sigma (\textbf{W}_1^{(l)} \textbf{a}^{l-1} + \textbf{b}_1^{(l)}; \omega _0, s_0) \cdot \\ e^{-|s_0(\textbf{W}_2^{(l)} \textbf{a}^{l-1} + \textbf{b}_2^{(l)})|^2}, \end{aligned} \end{aligned}$$

where $\sigma (x; \omega _0, s_0) = e^{j\omega _0 x} e^{-|s_0 x|^2}$, in which $\omega _0$ and $s_0$ control the frequency and scale of the complex Gabor wavelet. In that case, $\theta _l=\{\textbf{W}_1^{(l)},\textbf{b}_1^{(l)},\textbf{W}_2^{(l)},\textbf{b}_2^{(l)}\}$. The output layer is defined as

A2 $$\begin{aligned} \psi _{\theta '}(\textbf{a}^{L}) = \textbf{W}' \textbf{a}^{(L)} + \textbf{b}' \end{aligned}$$

with parameters $$\theta ' = \{\textbf{W}', \textbf{b}'\}$$.

#### SIREN.

SIREN [4] is a multilayer perceptron (MLP) with sinusoidal activations, demonstrated to effectively represent complex and high-frequency image structures. For the SIREN network, each hidden layer is defined as

A3 $$\begin{aligned} \phi _{\theta _l}^{(l)}(\textbf{a}^{(l-1)}) = \sigma _l(\textbf{W}^{(l)} \textbf{a}^{(l-1)} + \textbf{b}^{(l)}), \end{aligned}$$

where $\theta _l = \{\textbf{W}^{(l)}, \textbf{b}^{(l)}\}$ and $\sigma _l$ denotes the activation function at layer *l*. To capture high-frequency variations, the activation functions are chosen as

A4 $$\begin{aligned} \sigma _1(z) = \sin (w_0 z), \quad \sigma _l(z) = \sin (z), \, l \ge 2, \end{aligned}$$

where $w_0$ is a scaling constant applied to the first layer to enhance the representation of fine details. Finally, the output layer is defined as

A5 $$\begin{aligned} \psi _{\theta '}(\textbf{a}^{L}) = \textbf{W}' \textbf{a}^{(L)} + \textbf{b}' \end{aligned}$$

with parameters $$\theta ' = \{\textbf{W}', \textbf{b}'\}$$.

### Closed-form Expressions of Proximal Operators

In the proximal update of Eq. (8), each element of $\textbf{U}$ is updated via the proximal operator of Φ. To clarify this step and to facilitate implementation, we provide the explicit closed-form expressions of the proximal mappings corresponding to common sparse regularizers used in our framework. For a scalar variable $v \in \mathbb {R}$, the proximal mapping associated with Φ is defined as

A6 $$\begin{aligned} \operatorname {prox}_{\frac{\gamma \lambda }{\beta }\Phi }(v) {:}{=}\arg \min _{u \in \mathbb {R}} \frac{1}{2}(u-v)^2 + \frac{\gamma \lambda }{\beta }\Phi (u). \end{aligned}$$

*Note.* The proximal mapping is, in general, set-valued. In practice, we use its standard single-valued form (e.g., hard or soft thresholding) to ensure deterministic element-wise updates of $\textbf{U}$.

We next summarize the closed-form proximal mappings for three commonly used regularizers—$\ell _0$, $\ell _1$, and MCP—which correspond to hard, soft, and firm thresholding, respectively. These operators are essential in our sparse regularization module and are applied element-wise to the tensor $\textbf{U}$ during each iteration of the optimization.

*(a)* $\ell _0$ *regularization (hard thresholding):*

A7 $$\begin{aligned} \Phi (u) = \mathbb {I}[u \ne 0], \qquad \operatorname {prox}_{\frac{\gamma \lambda }{\beta }\Phi }(v) = {\left\{ \begin{array}{ll} 0, &  |v| \le \sqrt{\frac{2\gamma \lambda }{\beta }}, \\ v, &  |v| > \sqrt{\frac{2\gamma \lambda }{\beta }}. \end{array}\right. } \end{aligned}$$

*(b)* $\ell _1$ *regularization (soft thresholding):*

A8 $$\begin{aligned} \Phi (u) = |u|, \qquad \operatorname {prox}_{\frac{\gamma \lambda }{\beta }\Phi }(v) = \operatorname {sign}(v)\max \{|v| - \frac{\gamma \lambda }{\beta },0\}. \end{aligned}$$

*(c) MCP regularization (firm thresholding):* With shape parameter $\gamma _\text {shape} > 0$,

A9 $$\begin{aligned} \Phi (u) = {\left\{ \begin{array}{ll} |u| - \dfrac{u^2}{2\gamma _\text {shape}}, &  |u| \le \gamma _\text {shape},\\ \dfrac{\gamma _\text {shape}}{2}, &  |u| > \gamma _\text {shape}, \end{array}\right. } \end{aligned}$$

and the corresponding proximal operator is

A10 $$\begin{aligned} \operatorname {prox}_{\frac{\gamma \lambda }{\beta }\Phi }(v) = {\left\{ \begin{array}{ll} 0, &  |v| \le \frac{\gamma \lambda }{\beta }\\ \dfrac{\operatorname {sign}(v),(|v| - \frac{\gamma \lambda }{\beta })}{1 - (\frac{\gamma \lambda }{\beta })/\gamma _\text {shape}}, &  \frac{\gamma \lambda }{\beta } < |v| \le \gamma _\text {shape},\\ v, &  |v| > \gamma _\text {shape}, \end{array}\right. } \end{aligned}$$

where $\frac{\gamma \lambda }{\beta } < \gamma _\text {shape}$ ensures $1 - (\frac{\gamma \lambda }{\beta })/\gamma _\text {shape} > 0$. More detailed description on the proximity operator of the MCP can be found in [50].

For a tensor input $\textbf{V}$ of the same dimension as $\textbf{U}$, the proximal mappings of $\ell _0$, $\ell _1$, and MCP are applied element-wise, so that each spatial position and channel in $\textbf{V}$ is processed independently. This element-wise property allows efficient and parallel computation of the updates during optimization.

## Analysis of MG-SpaIR Algorithm

We first establish a necessary condition characterizing all global minimizers of Eq. (5) under the assumptions that $\mathcal {L}_\text {fid}$ is squared loss, Φ is $\ell _0$ regularization, and $\operatorname {OptStep}$ is a gradient descent update with step size η. Building on this condition, we derive a proximal gradient update tailored to the model’s sparsity structure and prove its convergence. Finally, we extend these results to the multi-grade setting to characterize the convergence properties of Algorithm 1.

### Characterizing Global Minimizers: A Necessary Condition

We first derive a necessary condition for the global minimizers of Eq. (5) under a single-grade setting. Consider a neural network with *L* hidden layers. For convenience, we vectorize the auxiliary variable $\textbf{U}$, the network parameters θ, and the network output $f_{\theta }(\textbf{X}_{\text {high}})$.

Define

B11 $$\begin{aligned} \begin{aligned} d_1&{:}{=} H_{\text {high}} W_{\text {high}} (2C),\\ d_2&{:}{=} \text {(number of trainable parameters)},\\ d_3&{:}{=} H_{\text {high}} W_{\text {high}} C. \end{aligned} \end{aligned}$$

Let $\boldsymbol{u}\in \mathbb {R}^{d_1}$ be the vectorized form of $\textbf{U}$ obtained by stacking all tensor columns. For each layer *l*, let $\textbf{W}_l$ and $\textbf{b}_l$ denote the weight matrix and bias vector, and let $\boldsymbol{W}_l$ and $\boldsymbol{b}_l$ be their vectorized forms. The full network parameter vector is

B12 $$\begin{aligned} \boldsymbol{\theta }=(\boldsymbol{W}_1^T,\boldsymbol{b}_1^T,\ldots ,\boldsymbol{W}_L^T,\boldsymbol{b}_L^T)^T\in \mathbb {R}^{d_2}. \end{aligned}$$

The output $f_{\theta }(\textbf{X}_{\text {high}})$ is likewise vectorized into $\boldsymbol{f}(\boldsymbol{\theta })\in \mathbb {R}^{d_3}$.

Because $\textbf{M}$, $\mathcal {S}_s$, $\mathcal {A}$, and $\mathcal {B}$ are linear operators, there exist matrices $\textbf{A}$ and $\textbf{B}$ such that $\textbf{A}\boldsymbol{f}(\boldsymbol{\theta })$ and $\textbf{B}\boldsymbol{f}(\boldsymbol{\theta })$ correspond to the vectorized forms of $\textbf{M}\odot (\mathcal {S}_s\mathcal {A})$ and $\mathcal {B}$ applied to the network output. Let $\boldsymbol{g}$ be the vectorized $\tilde{\textbf{Y}}$. Then Eq. (5) is equivalent to

B13 $$\begin{aligned} \begin{aligned} \min _{(\boldsymbol{u}, \boldsymbol{\theta }) \in \mathbb {R}^{d_1} \times \mathbb {R}^{d_2}}&\mathcal {L}(\boldsymbol{u}, \boldsymbol{\theta }){:}{=}\frac{1}{2}\Vert \boldsymbol{g} - \textbf{A}\boldsymbol{f}(\boldsymbol{\theta })\Vert _2^2 \\&+ \frac{\beta }{2}\Vert \boldsymbol{u} - \textbf{B}\boldsymbol{f}(\boldsymbol{\theta })\Vert _2^2 + \lambda \Vert \boldsymbol{u}\Vert _0 \end{aligned}, \end{aligned}$$

where $\Vert \cdot \Vert _2$ denotes the Euclidean norm and $\Vert \cdot \Vert _0$ the $\ell _0$ norm. For a proper, lower semi-continuous function $h: \mathbb {R}^{d_2} \rightarrow \mathbb {R}\cup \{\infty \}$ and γ>0, the proximal operator of *h* at $\boldsymbol{u}\in \mathbb {R}^{d_1}$ is

$$ \textrm{prox}_{\gamma h}(\boldsymbol{u}) {:}{=} \textrm{argmin}_{\boldsymbol{v}\in \mathbb {R}^{d_1}} \left\{ \frac{1}{2\gamma }\Vert \boldsymbol{v}-\boldsymbol{u}\Vert _2^2 + h(\boldsymbol{v})\right\} . $$

The following lemma is instrumental for characterizing the global minimizers of Eq. (B13).

#### Lemma B.1

For $$\gamma \in (0,1]$$ and $$\boldsymbol{a},\boldsymbol{b} \in \mathbb {R}^{d_1}$$,

B14 $$\begin{aligned} \frac{1}{\gamma }\Vert \gamma \boldsymbol{a}\Vert _2^2 - \Vert \boldsymbol{a}\Vert _2^2 \,\le \, \frac{1}{\gamma }\Vert \boldsymbol{b} - (1-\gamma )\boldsymbol{a}\Vert _2^2 - \Vert \boldsymbol{b}\Vert _2^2. \end{aligned}$$

#### Proof

The left-hand side equals $(\gamma -1)\Vert \boldsymbol{a}\Vert _2^2$. Thus, it suffices to show

B15 $$\begin{aligned} \Vert \boldsymbol{b} - (1-\gamma )\boldsymbol{a}\Vert _2^2 - \gamma \Vert \boldsymbol{b}\Vert _2^2 - \gamma (\gamma -1)\Vert \boldsymbol{a}\Vert _2^2 \ge 0. \end{aligned}$$

Expanding the squares using the inner product $\langle \cdot ,\cdot \rangle$ on $\mathbb {R}^{d_1}$ and combining similar terms leads to

B16 $$\begin{aligned} \begin{aligned}&\Vert \boldsymbol{b} - (1-\gamma )\boldsymbol{a}\Vert _2^2 - \gamma \Vert \boldsymbol{b}\Vert _2^2 - \gamma (\gamma -1)\Vert \boldsymbol{a}\Vert _2^2\\&= (1-\gamma )(\Vert \boldsymbol{b}\Vert _2^2 - 2\langle \boldsymbol{b},\boldsymbol{a}\rangle + \Vert \boldsymbol{a}\Vert _2^2) \\&= (1-\gamma )\Vert \boldsymbol{b}-\boldsymbol{a}\Vert _2^2 \,\ge \, 0, \end{aligned} \end{aligned}$$

since $\gamma \in (0,1]$. This proves Eq. (B14). □

Using the lemma above, we now establish a necessary condition for the global minimizers of Eq. (B13).

#### Theorem B.2

If the activation function of the neural network is differentiable, and let $\lambda ,\beta >0$, and if $(\boldsymbol{u}^*, \boldsymbol{\theta }^*) \in \mathbb {R}^{d_1}\times \mathbb {R}^{d_2}$ is a global minimizer of Eq. (B13), then for all $\gamma \in (0,1]$,

B17 $$\begin{aligned} \begin{aligned}&\boldsymbol{u}^* \in \textrm{prox}_{\frac{\gamma \lambda }{\beta }\Vert \cdot \Vert _0}\!\left( \gamma \textbf{B}\boldsymbol{f}(\boldsymbol{\theta }^*) + (1-\gamma )\boldsymbol{u}^*\right) , \\&\frac{\partial \mathcal {L}}{\partial \boldsymbol{\theta }}(\boldsymbol{u}^*, \boldsymbol{\theta }^*) = 0. \end{aligned} \end{aligned}$$

#### Proof

Since $(\boldsymbol{u}^*,\theta ^*)$ is a global minimizer of Eq. (B13), we have

B18 $$\begin{aligned} \mathcal {L}(\boldsymbol{u}^*,\theta ^*) \le \mathcal {L}(\boldsymbol{u},\theta ) \quad \text {for all }(\boldsymbol{u},\theta )\in \mathbb {R}^{d_1}\times \mathbb {R}^{d_2}. \end{aligned}$$

The proof proceeds in two main steps.

**Step 1:** Proximal inclusion for $\boldsymbol{u}^*$. Fix $\theta =\theta ^*$ in Eq. (B18). Writing out the objective terms that depend on $\boldsymbol{u}$ yields

$$ \frac{\beta }{2}\Vert \boldsymbol{u}^*-{\textbf{B}}\boldsymbol{f}(\theta ^*)\Vert _2^2 + \lambda \Vert \boldsymbol{u}^*\Vert _0 \le \frac{\beta }{2}\Vert \boldsymbol{u}-{\textbf{B}}\boldsymbol{f}(\theta ^*)\Vert _2^2 + \lambda \Vert \boldsymbol{u}\Vert _0, $$

for all $\boldsymbol{u}\in \mathbb {R}^{d_1}$. Since λ>0,

B19 $$\begin{aligned}&\frac{\beta }{2\lambda }\Vert \boldsymbol{u}^*-{\textbf{B}}\boldsymbol{f}(\theta ^*)\Vert _2^2 + \Vert \boldsymbol{u}^*\Vert _0\le \nonumber \\&\quad \frac{\beta }{2\lambda }\Vert \boldsymbol{u}-{\textbf{B}}\boldsymbol{f}(\theta ^*)\Vert _2^2 + \Vert \boldsymbol{u}\Vert _0. \end{aligned}$$

Apply Lemma B.1 with

$$ \boldsymbol{a} {:}{=} \boldsymbol{u}^* - {\textbf{B}}\boldsymbol{f}(\theta ^*), \qquad \boldsymbol{b} {:}{=} \boldsymbol{u} - {\textbf{B}}\boldsymbol{f}(\theta ^*), $$

and with the same $\gamma \in (0,1]$ that appears in the theorem. Lemma B.1 gives

$$ \frac{1}{\gamma }\Vert \gamma \boldsymbol{a}\Vert _2^2 - \Vert \boldsymbol{a}\Vert _2^2 \le \frac{1}{\gamma }\Vert \boldsymbol{b} - (1-\gamma )\boldsymbol{a}\Vert _2^2 - \Vert \boldsymbol{b}\Vert _2^2. $$

Substituting the definitions of $\boldsymbol{a},\boldsymbol{b}$ and multiplying the whole inequality by $\tfrac{\beta }{2\lambda }$ yields

B20 $$\begin{aligned} \begin{aligned}&\frac{\beta }{2\gamma \lambda }\Vert \gamma (\boldsymbol{u}^*-{\textbf{B}}\boldsymbol{f}(\theta ^*))\Vert _2^2 - \frac{\beta }{2\lambda }\Vert \boldsymbol{u}^*-{\textbf{B}}\boldsymbol{f}(\theta ^*)\Vert _2^2\\&\le \frac{\beta }{2\gamma \lambda }\Vert \boldsymbol{u}-{\textbf{B}}\boldsymbol{f}(\theta ^*)-(1-\gamma )(\boldsymbol{u}^*-{\textbf{B}}\boldsymbol{f}(\theta ^*))\Vert _2^2\\&\quad - \frac{\beta }{2\lambda }\Vert \boldsymbol{u}-{\textbf{B}}\boldsymbol{f}(\theta ^*)\Vert _2^2. \end{aligned} \end{aligned}$$

Adding inequality Eq. (B19) to Eq. (B20), with the negative $\tfrac{\beta }{2\lambda }\Vert \cdot \Vert _2^2$ terms cancel, leads to

$$\begin{aligned} \begin{aligned}&\frac{\beta }{2\gamma \lambda }\Vert \gamma (\boldsymbol{u}^*-{\textbf{B}}\boldsymbol{f}(\theta ^*))\Vert _2^2 + \Vert \boldsymbol{u}^*\Vert _0\\&\le \frac{\beta }{2\gamma \lambda }\Vert \boldsymbol{u}-{\textbf{B}}\boldsymbol{f}(\theta ^*)-(1-\gamma )(\boldsymbol{u}^*-{\textbf{B}}\boldsymbol{f}(\theta ^*))\Vert _2^2 + \Vert \boldsymbol{u}\Vert _0, \end{aligned} \end{aligned}$$

for all $\boldsymbol{u} \in \mathbb {R}^{d_1}$. Rewriting the squared norms inside each argument gives

$$\begin{aligned} \begin{aligned}&\frac{\beta }{2\gamma \lambda }\Vert \boldsymbol{u}^* - \gamma {\textbf{B}}\boldsymbol{f}(\theta ^*) - (1-\gamma )\boldsymbol{u}^*\Vert _2^2 + \Vert \boldsymbol{u}^*\Vert _0\\&\le \frac{\beta }{2\gamma \lambda }\Vert \boldsymbol{u} - \gamma {\textbf{B}}\boldsymbol{f}(\theta ^*) - (1-\gamma )\boldsymbol{u}^*\Vert _2^2 + \Vert \boldsymbol{u}\Vert _0. \end{aligned} \end{aligned}$$

This inequality holds for every $\boldsymbol{u}\in \mathbb {R}^{d_1}$. Define

$$ \tau {:}{=} \frac{\gamma \lambda }{\beta } > 0 \qquad \text {so that}\qquad \frac{\beta }{2\gamma \lambda } = \frac{1}{2\tau }. $$

Then the last inequality is exactly the statement that $\boldsymbol{u}^*$ minimizes the function

$$ \boldsymbol{v} \mapsto \frac{1}{2\tau }\Vert \boldsymbol{v} - (\gamma {\textbf{B}}\boldsymbol{f}(\theta ^*) + (1-\gamma )\boldsymbol{u}^*)\Vert _2^2 + \Vert \boldsymbol{v}\Vert _0 $$

over $\boldsymbol{v}\in \mathbb {R}^{d_1}$. By the definition of the proximal operator (applied to $h(\cdot )=\Vert \cdot \Vert _0$), this yields the proximal inclusion

$$ \boldsymbol{u}^* \in \operatorname {prox}_{\frac{\gamma \lambda }{\beta }\Vert \cdot \Vert _0}\!\big (\gamma {\textbf{B}}\boldsymbol{f}(\theta ^*) + (1-\gamma )\boldsymbol{u}^*\big ). $$

**Step 2: Stationarity for** $\theta ^*$. Fix $\boldsymbol{u}=\boldsymbol{u}^*$ in Eq. (B18). Then

$$ \mathcal {L}(\boldsymbol{u}^*,\theta ^*) \le \mathcal {L}(\boldsymbol{u}^*,\theta ) \quad \text {for all }\theta \in \mathbb {R}^{d_2}, $$

so $\theta ^*$ is a global minimizer of the unconstrained differentiable function $\theta \mapsto \mathcal {L}(\boldsymbol{u}^*,\theta )$. The differentiability follows from the assumed differentiability of the activation (hence of $\boldsymbol{f}(\theta )$) and the linearity of ${\textbf{B}}$ and ${\textbf{A}}$; thus, $\mathcal {L}(\boldsymbol{u}^*,\cdot )$ is differentiable on $\mathbb {R}^{d_2}$. By Fermat’s rule for unconstrained differentiable minimization, any global minimizer must satisfy the first-order condition

$$ \frac{\partial \mathcal {L}}{\partial \theta }(\boldsymbol{u}^*,\theta ^*) = 0. $$

Combining Steps 1 and 2 establishes Eq. (B17) for every $\gamma \in (0,1]$, completing the proof. □

### Proximal Gradient Scheme

Motivated by Theorem B.2, we employ a proximal gradient scheme for solving Eq. (B13). The scheme alternates between updating the auxiliary variable $\boldsymbol{u}$ via a proximal step and updating the network parameters $\boldsymbol{\theta }$ via gradient descent:

B21 $$\begin{aligned} \begin{aligned}&\boldsymbol{u}^{k+1} \in \textrm{prox}_{\tfrac{\gamma \lambda }{\beta }\Vert \cdot \Vert _0}\!\Big (\gamma \textbf{B}\boldsymbol{f}(\boldsymbol{\theta }^k) + (1 - \gamma )\boldsymbol{u}^k\Big ),\\&\boldsymbol{\theta }^{k+1} {:}{=} \boldsymbol{\theta }^{k} - \eta \,\frac{\partial \mathcal {L}}{\partial \boldsymbol{\theta }}(\boldsymbol{u}^{k+1}, \boldsymbol{\theta }^k), \end{aligned} \end{aligned}$$

where η>0 is the step size for the gradient update of $\boldsymbol{\theta }$.

The proximal gradient scheme can be interpreted as a two-step iterative process:

1. The $\boldsymbol{u}$-update enforces sparsity via hard thresholding, promoting a concise representation of the auxiliary variable.
2. The $\boldsymbol{\theta }$-update adjusts the network parameters to reduce the overall loss, taking into account the current sparse approximation $\boldsymbol{u}^{k+1}$.

### Convergence Analysis

We analyze the convergence property of the proximal–gradient method in Eq. (B21). To ensure that all quantities involved in the analysis remain well defined, we assume the existence of a convex and compact set $\Omega \subset \mathbb {R}^{d_1} \times \mathbb {R}^{d_2}$ and some $\eta _0 > 0$ such that both sequences ${(\boldsymbol{u}^k, \theta ^k)}_{k=0}^\infty$ and ${(\boldsymbol{u}^{k+1}, \theta ^k)}_{k=0}^\infty$ generated by the algorithm remain in Ω for all step sizes $\eta \in (0,\eta _0)$. This boundedness assumption ensures, in particular, that the loss landscape is explored only within a compact region.

Since the neural network activation function is twice continuously differentiable, the mapping $\theta \mapsto \mathcal {L}(\boldsymbol{u},\theta )$ is twice continuously differentiable for every fixed $\boldsymbol{u}$. Let $\boldsymbol{H}_{\mathcal {L}}(\boldsymbol{u},\theta )$ denote the Hessian of $\mathcal {L}$ with respect to θ, and define

B22 $$\begin{aligned} \alpha {:}{=} \sup \bigl \{\Vert \boldsymbol{H}_\mathcal {L}(\boldsymbol{u}, \theta )\Vert _2 : (\boldsymbol{u}, \theta ) \in \Omega \bigr \}, \end{aligned}$$

where $\Vert \cdot \Vert _2$ is the spectral norm. The finiteness of α follows from the compactness of Ω.

The next lemma establishes a key descent property for the proximal–gradient update and serves as the main building block of the convergence proof.

#### Lemma B.3

Let $\{(\boldsymbol{u}^k, \theta ^k)\}_{k=0}^\infty$ be generated by Eq. (B21) with initial point $(\boldsymbol{u}^0, \theta ^0)$. Then for all k≥1,

B23 $$\begin{aligned} \mathcal {L}(\boldsymbol{u}^{k+1}, \theta ^k) + \frac{\beta }{2}\frac{1-\gamma }{\gamma }\Vert \boldsymbol{u}^{k+1} - \boldsymbol{u}^k\Vert _2^2 \le \mathcal {L}(\boldsymbol{u}^k, \theta ^k). \end{aligned}$$

#### Proof

By the definition of proximal operators, the first update in Eq. (B21) is equivalent to

B24 $$\begin{aligned} \boldsymbol{u}^{k+1} \in&\textrm{argmin}_{\boldsymbol{u} \in \mathbb {R}^{d_1}} \{ \tfrac{1}{2}\tfrac{\beta }{\gamma \lambda } \Vert \boldsymbol{u} - \gamma \boldsymbol{B}\boldsymbol{f}(\theta ^k) - (1-\gamma )\boldsymbol{u}^k\Vert _2^2\nonumber \\&\qquad \qquad \qquad + \Vert \boldsymbol{u}\Vert _0\}. \end{aligned}$$

Expanding the quadratic term yields

B25 $$\begin{aligned} \begin{aligned}&\Vert \boldsymbol{u} - \gamma \boldsymbol{B}\boldsymbol{f}(\theta ^k) - (1-\gamma )\boldsymbol{u}^k\Vert _2^2\\&= \gamma \Vert \boldsymbol{u} - \boldsymbol{B}\boldsymbol{f}(\theta ^k)\Vert _2^2 + (1-\gamma )\Vert \boldsymbol{u} - \boldsymbol{u}^k\Vert _2^2 + r, \end{aligned} \end{aligned}$$

where *r* is independent of $\boldsymbol{u}$ and thus irrelevant to minimization. Substituting back, we obtain

B26 $$\begin{aligned}&\tfrac{1}{2}\tfrac{\beta }{\gamma \lambda }\Vert \boldsymbol{u} - \gamma \boldsymbol{B}\boldsymbol{f}(\theta ^k) - (1-\gamma )\boldsymbol{u}^k\Vert _2^2 + \Vert \boldsymbol{u}\Vert _0\nonumber \\&= \tfrac{1}{\lambda }\left( \tfrac{\beta }{2}\Vert \boldsymbol{u} - \boldsymbol{B}\boldsymbol{f}(\theta ^k)\Vert _2^2 + \lambda \Vert \boldsymbol{u}\Vert _0 + \tfrac{\beta }{2}\tfrac{1-\gamma }{\gamma }\Vert \boldsymbol{u} - \boldsymbol{u}^k\Vert _2^2 \right) \nonumber \\&\quad + c, \end{aligned}$$

with *c* again independent of $\boldsymbol{u}$. By the definition of $\mathcal {L}$, this is equivalent to

B27 $$\begin{aligned} \boldsymbol{u}^{k+1} \in \textrm{argmin}_{\boldsymbol{u} \in \mathbb {R}^{d_1}} \left\{ \mathcal {L}(\boldsymbol{u}, \theta ^k) + \tfrac{\beta }{2}\tfrac{1-\gamma }{\gamma }\Vert \boldsymbol{u} - \boldsymbol{u}^k\Vert _2^2 \right\} . \end{aligned}$$

Hence, $$\boldsymbol{u}^{k+1}$$ satisfies

B28 $$\begin{aligned} \begin{aligned}&\mathcal {L}(\boldsymbol{u}^{k+1}, \theta ^k) + \tfrac{\beta }{2}\tfrac{1-\gamma }{\gamma }\Vert \boldsymbol{u}^{k+1} - \boldsymbol{u}^k\Vert _2^2\\&\le \mathcal {L}(\boldsymbol{u}, \theta ^k) + \tfrac{\beta }{2}\tfrac{1-\gamma }{\gamma }\Vert \boldsymbol{u} - \boldsymbol{u}^k\Vert _2^2 \end{aligned} \end{aligned}$$

for all $\boldsymbol{u} \in \mathbb {R}^{d_1}$. Taking $\boldsymbol{u} = \boldsymbol{u}^k$ gives the claim. □

The following convergence theorem holds under these assumptions.

#### Theorem B.4

Let $\{(\boldsymbol{u}^k, \theta ^k)\}_{k=0}^\infty$ be the sequence generated by the proximal gradient method Eq. (B21) with initial guess $(\boldsymbol{u}^0, \theta ^0)$. Suppose the activation function in the neural network is twice continuously differentiable, and there exists a convex, compact set $\Omega \subset \mathbb {R}^{d_1} \times \mathbb {R}^{d_2}$ such that

$$ \{(\boldsymbol{u}^k, \theta ^k)\}_{k=0}^\infty \subset \Omega , \quad \{(\boldsymbol{u}^{k+1}, \theta ^k)\}_{k=0}^\infty \subset \Omega . $$

Assume that α defined in Eq. (B22) is finite. If the learning rate $\eta \in (0, 2/\alpha )$ and the parameter $\gamma \in (0,1)$, then the following hold:

1. $$\displaystyle \lim _{k\rightarrow \infty }\mathcal {L}(\boldsymbol{u}^k, \theta ^k) = L^*$$ for some $$L^* \ge 0$$;
2. $$\displaystyle \lim _{k\rightarrow \infty }\Vert \boldsymbol{u}^{k+1} - \boldsymbol{u}^k\Vert _2 = 0,$$ and $$\displaystyle \lim _{k\rightarrow \infty }\Vert \theta ^{k+1} - \theta ^k\Vert _2 = 0$$;
3. $$\displaystyle \lim _{k\rightarrow \infty }\textrm{dist}\!\left( \boldsymbol{0}, \tfrac{\partial \mathcal {L}}{\partial \boldsymbol{u}}(\boldsymbol{u}^{k+1}, \theta ^k)\right) \,\mathord {=}\, 0,$$ $$\text {and }\displaystyle \lim _{k\rightarrow \infty }\tfrac{\partial \mathcal {L}}{\partial \theta }(\boldsymbol{u}^{k+1}, \theta ^k)$$ = 0.

Here $\tfrac{\partial \mathcal {L}}{\partial \boldsymbol{u}}(\boldsymbol{u}^{k+1}, \theta ^k)$ denotes the subdifferential of $\mathcal {L}(\cdot , \theta ^k)$ at $\boldsymbol{u}^{k+1}$, and $\tfrac{\partial \mathcal {L}}{\partial \theta }(\boldsymbol{u}^{k+1}, \cdot )$ is the gradient with respect to θ at $\theta ^k$. The distance between a point $\boldsymbol{p}\in \mathbb {R}^{d_1}$ and a set $S\subset \mathbb {R}^{d_1}$ is defined as

$$ \textrm{dist}(\boldsymbol{p}, S) {:}{=} \inf \{\Vert \boldsymbol{q}-\boldsymbol{p}\Vert _2 : \boldsymbol{q}\in S\}. $$

#### Proof

Since the activation function is twice continuously differentiable, $\mathcal {L}(\boldsymbol{u}, \theta )$ is twice continuously differentiable with respect to θ. By the Taylor expansion, there exists some $\bar{\theta }^k$ between $\theta ^k$ and $\theta ^{k+1}$ such that

B29 $$\begin{aligned} \begin{aligned}&\mathcal {L}(\boldsymbol{u}^{k+1}, \theta ^{k+1})\\&= \mathcal {L}(\boldsymbol{u}^{k+1}, \theta ^k) + \frac{\partial \mathcal {L}}{\partial \theta }(\boldsymbol{u}^{k+1}, \theta ^k)^T (\theta ^{k+1} - \theta ^k)\\&\quad + \frac{1}{2} (\theta ^{k+1} - \theta ^k)^T \boldsymbol{H}_\mathcal {L}(\boldsymbol{u}^{k+1}, \bar{\theta }^k) (\theta ^{k+1} - \theta ^k). \end{aligned} \end{aligned}$$

From the second step of Eq. (B21), we have

B30 $$\begin{aligned} \frac{\partial \mathcal {L}}{\partial \theta }(\boldsymbol{u}^{k+1}, \theta ^k) = -\frac{1}{\eta } (\theta ^{k+1} - \theta ^k). \end{aligned}$$

Substituting this into Eq. (B29) gives

B31 $$\begin{aligned} \begin{aligned}&\mathcal {L}(\boldsymbol{u}^{k+1}, \theta ^{k+1})\\&= \mathcal {L}(\boldsymbol{u}^{k+1}, \theta ^k) - \frac{1}{\eta }\Vert \theta ^{k+1} - \theta ^k\Vert _2^2\\&\quad + \frac{1}{2} (\theta ^{k+1} - \theta ^k)^T \boldsymbol{H}_\mathcal {L}(\boldsymbol{u}^{k+1}, \bar{\theta }^k) (\theta ^{k+1} - \theta ^k). \end{aligned} \end{aligned}$$

By convexity of Ω and the assumption that $(\boldsymbol{u}^k, \theta ^k), \ (\boldsymbol{u}^{k+1}, \theta ^k) \in \Omega$, we have $(\boldsymbol{u}^{k+1}, \bar{\theta }^k) \in \Omega$. Using the spectral norm bound Eq. (B22) yields

B32 $$\begin{aligned} \begin{aligned}&\left| \frac{1}{2} (\theta ^{k+1} - \theta ^k)^T \boldsymbol{H}_\mathcal {L}(\boldsymbol{u}^{k+1}, \bar{\theta }^k) (\theta ^{k+1} - \theta ^k) \right| \\&\le \frac{\alpha }{2} \Vert \theta ^{k+1} - \theta ^k\Vert _2^2, \end{aligned} \end{aligned}$$

which substituted into Eq. (B31) gives

B33 $$\begin{aligned}&\mathcal {L}(\boldsymbol{u}^{k+1}, \theta ^{k+1}) \le \mathcal {L}(\boldsymbol{u}^{k+1}, \theta ^k) \nonumber \\&\quad + \left( \frac{\alpha }{2} - \frac{1}{\eta } \right) \Vert \theta ^{k+1} - \theta ^k\Vert _2^2. \end{aligned}$$

From Lemma B.3, we also have

B34 $$\begin{aligned} \mathcal {L}(\boldsymbol{u}^{k+1}, \theta ^k) + \frac{\beta }{2}\frac{1-\gamma }{\gamma } \Vert \boldsymbol{u}^{k+1} - \boldsymbol{u}^k\Vert _2^2 \le \mathcal {L}(\boldsymbol{u}^k, \theta ^k). \end{aligned}$$

Combining Eq. (B33) and Eq. (B34) leads to

B35 $$\begin{aligned} \begin{aligned} \mathcal {L}(\boldsymbol{u}^{k+1}, \theta ^{k+1})&+ \frac{\beta }{2}\frac{1-\gamma }{\gamma } \Vert \boldsymbol{u}^{k+1} - \boldsymbol{u}^k\Vert _2^2\\&+ \left( \frac{1}{\eta } - \frac{\alpha }{2} \right) \Vert \theta ^{k+1} - \theta ^k\Vert _2^2\\&\le \mathcal {L}(\boldsymbol{u}^k, \theta ^k). \end{aligned} \end{aligned}$$

Since $\frac{\beta }{2}\frac{1-\gamma }{\gamma } > 0$ and $\frac{1}{\eta } - \frac{\alpha }{2} > 0$, we conclude that $\{\mathcal {L}(\boldsymbol{u}^k, \theta ^k)\}$ is monotonically non-increasing and bounded below by zero. Hence, there exists $L^* \ge 0$ such that

B36 $$\begin{aligned} \lim _{k \rightarrow \infty } \mathcal {L}(\boldsymbol{u}^k, \theta ^k) = L^*, \end{aligned}$$

proving Item 1.

Equation Eq. (B35) also implies

B37 $$\begin{aligned} \lim _{k \rightarrow \infty } \Vert \boldsymbol{u}^{k+1} - \boldsymbol{u}^k\Vert _2 = 0, \quad \lim _{k \rightarrow \infty } \Vert \theta ^{k+1} - \theta ^k\Vert _2 = 0, \end{aligned}$$

which proves Item 2.

For Item 3, from Eq. (B27) we have

B38 $$\begin{aligned} -\frac{\beta (1-\gamma )}{\gamma } (\boldsymbol{u}^{k+1} - \boldsymbol{u}^k) \in \frac{\partial \mathcal {L}}{\partial \boldsymbol{u}}(\boldsymbol{u}^{k+1}, \theta ^k), \end{aligned}$$

so

B39 $$\begin{aligned} \textrm{dist}\left( \boldsymbol{0}, \frac{\partial \mathcal {L}}{\partial \boldsymbol{u}}(\boldsymbol{u}^{k+1}, \theta ^k)\right) \le \frac{\beta (1-\gamma )}{\gamma } \Vert \boldsymbol{u}^{k+1} - \boldsymbol{u}^k\Vert _2 \rightarrow 0.\nonumber \\ \end{aligned}$$

Similarly, the gradient update gives

B40 $$\begin{aligned} \frac{\partial \mathcal {L}}{\partial \theta }(\boldsymbol{u}^{k+1}, \theta ^k) = -\frac{1}{\eta } (\theta ^{k+1} - \theta ^k) \rightarrow 0, \end{aligned}$$

proving Item 3 and completing the proof. □

### Extension to the Multi-Grade Setting

Let $\boldsymbol{\theta }_l$ denote the vectorized parameters of $\theta ^{(l)}$, and let $\boldsymbol{z}_l(\boldsymbol{\theta }_l)$ denote the vectorized network output $f^{(l)}_{\theta ^{(l)};\Theta _{\le l-1}^*}({\textbf{X}}_{\text {high}})$ at grade *l*. Note that the lower-grade parameters $\Theta ^{*}_{\le l-1}$ are fixed during grade-*l* optimization and enter $\mathcal {L}_l$ only as constants. Hence, they do not affect the Lipschitz or smoothness properties of the $\theta _l$-gradient. Let $d_{2,l}$ be the dimension of $\boldsymbol{\theta }_l$. The vectorized optimization model in grade *l* is

B41 $$\begin{aligned} \begin{aligned} \min _{(\boldsymbol{u}, \boldsymbol{\theta }_l) \in \mathbb {R}^{d_1} \times \mathbb {R}^{d_{2,l}}}&\mathcal {L}_l(\boldsymbol{u}, \boldsymbol{\theta }_l) {:}{=} \frac{1}{2}\Vert \boldsymbol{g} - \textbf{A}\boldsymbol{z}_l(\boldsymbol{\theta }_l)\Vert _2^2\\&+ \frac{\beta }{2}\Vert \boldsymbol{u} - \textbf{B}\boldsymbol{z}_l(\boldsymbol{\theta }_l)\Vert _2^2 + \lambda \Vert \boldsymbol{u}\Vert _0. \end{aligned} \end{aligned}$$

Theorem B.2 implies that if a pair $(\boldsymbol{u}_l^*, \boldsymbol{\theta }_l^*) \in \mathbb {R}^{d_1} \times \mathbb {R}^{d_{2,l}}$ is a global minimizer of Eq. (B41), then there holds that

$$\begin{aligned}&\boldsymbol{u}_l^* \in \textrm{prox}_{\frac{\gamma \lambda }{\beta }\Vert \cdot \Vert _0}(\gamma \textbf{B} \boldsymbol{z}_l(\boldsymbol{\theta }_l^*) + (1-\gamma ) \boldsymbol{u}_l^*),\\&\quad \frac{\partial \mathcal {L}_l}{\partial \boldsymbol{\theta }_l}(\boldsymbol{u}_l^*, \boldsymbol{\theta }_l^*) = 0, \end{aligned}$$

for $\gamma \in (0,1]$. The proximal gradient updates in grade *l* are

$$\begin{aligned} \boldsymbol{u}_l^{k+1}&\in \textrm{prox}_{\frac{\gamma _l \lambda }{\beta }\Vert \cdot \Vert _0}(\gamma _l \textbf{B} \boldsymbol{z}_l(\boldsymbol{\theta }_l^k) \\&\quad + (1-\gamma _l) \boldsymbol{u}_l^k),\\ \boldsymbol{\theta }_l^{k+1}&{:}{=} \boldsymbol{\theta }_l^k - \eta _l \frac{\partial \mathcal {L}_l}{\partial \boldsymbol{\theta }_l}(\boldsymbol{u}_l^{k+1}, \boldsymbol{\theta }_l^k), \end{aligned}$$

with initial point $(\boldsymbol{u}_l^0, \boldsymbol{\theta }_l^0)$.

Assume the existence of a convex and compact set $\Omega _l \subset \mathbb {R}^{d_1} \times \mathbb {R}^{d_{2,l}}$ and $\eta _0 > 0$ such that

$$ \{(\boldsymbol{u}_l^k, \boldsymbol{\theta }_l^k)\}_{k=0}^\infty \subset \Omega _l, \quad \{(\boldsymbol{u}_l^{k+1}, \boldsymbol{\theta }_l^k)\}_{k=0}^\infty \subset \Omega _l, $$

for all $\eta \in (0, \eta _0)$ and define

$$ \alpha _l {:}{=} \sup \{\Vert \boldsymbol{H}_{\mathcal {L}_l}(\boldsymbol{u}, \boldsymbol{\theta }_l)\Vert _2 : (\boldsymbol{u}, \boldsymbol{\theta }_l) \in \Omega _l\}. $$

The finiteness of $\alpha _l$ follows from the twice continuous differentiability of the activation functions and the compactness of $\Omega _l$, which together ensure that $H_{\mathcal {L}_l}$ is continuous and therefore bounded on $\Omega _l$. Since the grade-*l* update in Eq. (B41) has exactly the same proximal–gradient structure as Eq. (B21), the assumptions of Theorem B.4 hold for each grade with the corresponding $\Omega _l$ and $\alpha _l$. Consequently, the convergence theorem for the multi-grade setting follows directly.

#### Theorem B.5

Let $\{(\boldsymbol{u}_l^k, \boldsymbol{\theta }_l^k)\}_{k=0}^\infty$ be generated by Eq. (B41) with initial guess $(\boldsymbol{u}_l^0, \boldsymbol{\theta }_l^0)$. Assume the activation function is twice continuously differentiable, $\Omega _l$ exists as above, and $\alpha _l < \infty$. If $\eta _l \in (0, 2/\alpha _l)$ and $\gamma _l \in (0,1)$, then

1. $$\displaystyle \lim _{k\rightarrow \infty } \mathcal {L}_l(\boldsymbol{u}_l^k, \boldsymbol{\theta }_l^k) = L_l^* \ge 0$$;
2. $$\displaystyle \lim _{k\rightarrow \infty } \Vert \boldsymbol{u}_l^{k+1}-\boldsymbol{u}_l^k\Vert _2 = 0,$$ and $$\displaystyle \lim _{k\rightarrow \infty } \Vert \boldsymbol{\theta }_l^{k+1}-\boldsymbol{\theta }_l^k\Vert _2 = 0$$;
3. $$\displaystyle \lim _{k\rightarrow \infty } \textrm{dist}\left( \boldsymbol{0}, \frac{\partial \mathcal {L}_l}{\partial \boldsymbol{u}_l}(\boldsymbol{u}_l^{k+1}, \boldsymbol{\theta }_l^k)\right) \, \mathord {=} \, 0,$$ $$\text { and } \displaystyle \lim _{k\rightarrow \infty } \frac{\partial \mathcal {L}_l}{\partial \boldsymbol{\theta }_l}(\boldsymbol{u}_l^{k+1}, \boldsymbol{\theta }_l^k) = 0$$.

Here $\tfrac{\partial \mathcal {L}}{\partial \boldsymbol{u}_l}(\boldsymbol{u}_l^{k+1}, \theta _l^k)$ denotes the subdifferential of $\mathcal {L}(\cdot , \theta _l^k)$ at $\boldsymbol{u}_l^{k+1}$, and $\tfrac{\partial \mathcal {L}}{\partial \theta _l}(\boldsymbol{u}_l^{k+1}, \cdot )$ is the gradient with respect to $\theta _l$ at $\theta _l^k$. The distance between a point $\boldsymbol{p}\in \mathbb {R}^{d_1}$ and a set $S\subset \mathbb {R}^{d_1}$ is defined as

$$ \textrm{dist}(\boldsymbol{p}, S) {:}{=} \inf \{\Vert \boldsymbol{q}-\boldsymbol{p}\Vert _2 : \boldsymbol{q}\in S\}. $$

Theorem 4.1 is merely a matrix-form rewriting of Theorem B.5, obtained by stacking the variables $\{(\boldsymbol{u}_l, \boldsymbol{\theta }_l)\}_l$ into $({\textbf{U}}, \theta )$. Hence, the result carries over directly.

### Spectral Analysis

This subsection derives a spectral stability condition for Algorithm 1. By linearizing the θ-gradient around the previous iterate, the update is rewritten as an affine recursion whose linear part is governed by the Hessian. This leads to the spectral quantity τ, which bounds the norm of the linearized update operator. When τ<1, the recursion becomes contractive, providing a sufficient condition that guarantees stable and convergent parameter dynamics.

Recall the updates from Eq. (B21):

$$\begin{aligned} \begin{aligned}&\boldsymbol{u}^{k+1} \in \textrm{prox}_{\tfrac{\gamma \lambda }{\beta }\Vert \cdot \Vert _0}\big (\gamma \boldsymbol{B}\boldsymbol{f}(\theta ^k) + (1 - \gamma )\boldsymbol{u}^k\big ),\\&\theta ^{k+1} {:}{=} \theta ^{k} - \eta \frac{\partial \mathcal {L}}{\partial \theta }(\boldsymbol{u}^{k + 1}, \theta ^k). \end{aligned} \end{aligned}$$

The second update is a gradient descent step in θ, but with the gradient evaluated at the freshly updated $\boldsymbol{u}^{k+1}$. To study convergence, we view this update as a fixed-point (Picard) iteration:

$$\begin{aligned} \theta ^{k+1} = \big (\mathcal {I} - \eta \,\tfrac{\partial \mathcal {L}}{\partial \theta }(\boldsymbol{u}^{k + 1}, \cdot )\big )(\theta ^k), \end{aligned}$$

where $\mathcal {I}$ denotes the identity operator. Classical Picard theory guarantees convergence when the update operator is non-expansive, whereas in deep networks the map

$$ \mathcal {I} - \eta \,\tfrac{\partial \mathcal {L}}{\partial \theta }(\boldsymbol{u}^{k + 1}, \cdot ) $$

can be expansive. To analyze the resulting dynamics, we therefore apply a Taylor expansion of the gradient around the previous iterate.

Expanding around $\theta ^{k-1}$, we approximate

B42 $$\begin{aligned} \begin{aligned}&\frac{\partial \mathcal {L}}{\partial \theta }(\boldsymbol{u}^{k + 1}, \theta ^k)\\&= \frac{\partial \mathcal {L}}{\partial \theta }(\boldsymbol{u}^{k + 1}, \theta ^{k - 1}) + \boldsymbol{H}_{\mathcal {L}}(\boldsymbol{u}^{k + 1}, \theta ^{k-1})(\theta ^k - \theta ^{k-1})\\&\quad + \tfrac{1}{2}(\theta ^k - \theta ^{k-1})^T\boldsymbol{T}_{\mathcal {L}}(\boldsymbol{u}^{k + 1}, \bar{\theta }^k)(\theta ^k - \theta ^{k-1}), \end{aligned} \end{aligned}$$

where $\boldsymbol{H}_{\mathcal {L}}$ and $\boldsymbol{T}_{\mathcal {L}}$ denote the Hessian and third-order derivative tensor of $\mathcal {L}$ with respect to θ, and $\bar{\theta }^k$ lies between $\theta ^{k-1}$ and $\theta ^k$.

Define

B43 $$\begin{aligned} {\left\{ \begin{array}{ll} \boldsymbol{v}^k {:}{=} -\eta \,\tfrac{\partial \mathcal {L}}{\partial \theta }(\boldsymbol{u}^{k + 1}, \theta ^{k - 1}) + \eta \,\boldsymbol{H}_{\mathcal {L}}(\boldsymbol{u}^{k + 1}, \theta ^{k - 1})\theta ^{k - 1},\\ \\ \boldsymbol{r}^k {:}{=} -\tfrac{\eta }{2}(\theta ^k - \theta ^{k-1})^T\boldsymbol{T}_{\mathcal {L}}(\boldsymbol{u}^{k + 1}, \bar{\theta }^k)(\theta ^k - \theta ^{k-1}),\\ \\ \boldsymbol{C}^k {:}{=} \boldsymbol{I} - \eta \,\boldsymbol{H}_{\mathcal {L}}(\boldsymbol{u}^{k + 1}, \theta ^{k - 1}), \end{array}\right. } \end{aligned}$$

with $\boldsymbol{I}$ being the identity. The θ-update can then be written as

B44 $$\begin{aligned} \theta ^{k+1} = \boldsymbol{C}^k\theta ^k + \boldsymbol{v}^k + \boldsymbol{r}^k. \end{aligned}$$

Let

B45 $$\begin{aligned} \tau {:}{=} \sup _{(\boldsymbol{u}, \theta ) \in \Omega }\Vert \boldsymbol{I} - \eta \boldsymbol{H}_{\mathcal {L}}(\boldsymbol{u}, \theta )\Vert _2, \end{aligned}$$

where $\Omega \subset \mathbb {R}^{d_1} \times \mathbb {R}^{d_2}$ is convex and compact and $\boldsymbol{I}$ is the identity matrix. The following result characterizes the convergence of $\{\theta ^k\}$.

#### Theorem B.6

Assume the activation function of the neural network is three times continuously differentiable, and let $\Omega \subset \mathbb {R}^{d_1} \times \mathbb {R}^{d_2}$ be a convex and compact set. Suppose the iterates $\{(\boldsymbol{u}^k, \theta ^k)\}_{k=1}^\infty$ are generated by Eq. (B21) and satisfy

$$ (\boldsymbol{u}^k, \theta ^k) \in \Omega ,\quad (\boldsymbol{u}^{k+1}, \theta ^k) \in \Omega ,\quad (\boldsymbol{u}^{k+1}, \theta ^{k-1}) \in \Omega $$

for all *k*. If τ<1, then the parameter sequence $\{\theta ^k\}_{k=1}^\infty$ converges.

#### Proof

From Eq. (B44), we have

B46 $$\begin{aligned} \begin{aligned} \theta ^{k+1}&= \Big (\prod _{j=1}^k \boldsymbol{C}^j\Big )\theta ^1 + \sum _{m=1}^k\Big (\prod _{j=m+1}^k \boldsymbol{C}^j\Big )\boldsymbol{v}^m\\&\quad + \sum _{m=1}^k\Big (\prod _{j=m+1}^k \boldsymbol{C}^j\Big )\boldsymbol{r}^m. \end{aligned} \end{aligned}$$

To prove convergence of $\{\theta ^k\}$, it suffices to show that each of the three terms on the right-hand side of Eq. (B46) converges.

**1. The homogeneous term.** By definition of τ, we have

$$ \Vert \boldsymbol{C}^k\Vert _2 \le \tau \ \ \text{ for } \text{ all }\ \ k. $$

Thus,

$$ \Big \Vert \Big (\prod _{j=1}^k \boldsymbol{C}^j\Big )\theta ^1\Big \Vert _2 \le \Vert \theta ^1\Vert _2 \prod _{j=1}^k \Vert \boldsymbol{C}^j\Vert _2 \le \tau ^k \Vert \theta ^1\Vert _2. $$

Since τ<1, it follows that $\tau ^k \rightarrow 0$ as $k \rightarrow \infty$, and hence,

$$ \lim _{k\rightarrow \infty }\Big \Vert \Big (\prod _{j=1}^k \boldsymbol{C}^j\Big )\theta ^1\Big \Vert _2 = 0. $$

Therefore, the first term converges to zero.

**2. The** $\boldsymbol{v}^m$ **term.** Because the activation is three times continuously differentiable, $\mathcal {L}(\boldsymbol{u},\theta )$ is smooth in θ. Although $\mathcal {L}$ is not continuous in $\boldsymbol{u}$ due to the $\Vert \cdot \Vert _0$ term, this term vanishes in differentiation with respect to θ, so both $\tfrac{\partial \mathcal {L}}{\partial \theta }$ and $\boldsymbol{H}_{\mathcal {L}}$ are continuous in $(\boldsymbol{u},\theta )$. Compactness of Ω ensures the existence of a constant V>0 such that

$$ \big \Vert -\eta \tfrac{\partial \mathcal {L}}{\partial \theta }(\boldsymbol{u},\theta ) + \eta \boldsymbol{H}_{\mathcal {L}}(\boldsymbol{u},\theta )\theta \big \Vert _2 \le V, \qquad \text{ for } \text{ all }\ \ (\boldsymbol{u},\theta )\in \Omega . $$

By the definition of $\boldsymbol{v}^k$, this implies

$$ \Vert \boldsymbol{v}^k\Vert _2 \le V, \ \ \text{ for } \text{ all }\ \ k. $$

Moreover,

$$ \Big \Vert \Big (\prod _{j=m+1}^k \boldsymbol{C}^j\Big )\boldsymbol{v}^m\Big \Vert _2 \le \Vert \boldsymbol{v}^m\Vert _2 \prod _{j=m+1}^k \Vert \boldsymbol{C}^j\Vert _2 \le V \tau ^{k-m}. $$

Hence,

$$\begin{aligned} \sum _{m=1}^k \Big \Vert \Big (\prod _{j=m+1}^k \boldsymbol{C}^j\Big )\boldsymbol{v}^m\Big \Vert _2&\le V \sum _{m=1}^k \tau ^{k-m}\\&= V \frac{1-\tau ^k}{1-\tau }\\&< \frac{V}{1-\tau }. \end{aligned}$$

This shows that the series

$$ \sum _{m=1}^\infty \left( \prod _{j=m+1}^\infty \boldsymbol{C}^j\right) \boldsymbol{v}^m $$

converges absolutely, and hence, the second term converges.

**3. The** $\boldsymbol{r}^m$ **term.** Since $\mathcal {L}(\boldsymbol{u},\theta )$ is three times continuously differentiable, $\boldsymbol{T}_{\mathcal {L}}(\boldsymbol{u},\theta )$ is continuous in $(\boldsymbol{u},\theta )$. By compactness of Ω, there exists T>0 such that $\Vert \boldsymbol{T}_{\mathcal {L}}(\boldsymbol{u},\theta )\Vert _2 \le T$ for all $(\boldsymbol{u},\theta )\in \Omega$. By convexity of Ω, the points $(\boldsymbol{u}^{k+1},\bar{\theta }^k)$ also lie in Ω, so

$$ \Vert \boldsymbol{T}_{\mathcal {L}}(\boldsymbol{u}^{k+1},\bar{\theta }^k)\Vert _2 \le T, \qquad \forall k. $$

By definition of $\boldsymbol{r}^k$, we obtain

$$\begin{aligned}&\Vert \boldsymbol{r}^k\Vert _2 \le \frac{\eta }{2}\Vert \boldsymbol{T}_{\mathcal {L}}(\boldsymbol{u}^{k+1},\bar{\theta }^k)\Vert _2 \Vert \theta ^k - \theta ^{k-1}\Vert _2^2 \le \frac{\eta T}{2}\Vert \theta ^k -\\&\quad \theta ^{k-1}\Vert _2^2. \end{aligned}$$

Since $\{(\boldsymbol{u}^k,\theta ^k)\}$ is bounded, there exists U>0 such that $\Vert \theta ^k - \theta ^{k-1}\Vert _2 \le U$ for all *k*. Therefore,

$$ \Vert \boldsymbol{r}^k\Vert _2 \le \frac{\eta T U^2}{2}, \qquad \forall k. $$

Following the same argument as for the $\boldsymbol{v}^m$ term, we conclude that

$$ \sum _{m=1}^\infty \left( \prod _{j=m+1}^\infty \boldsymbol{C}^j\right) \boldsymbol{r}^m $$

is absolutely convergent, and hence, the third term converges.

Since all three components in Eq. (B46) converge, the sequence $\{\theta ^k\}$ converges as claimed. □

## Experiment Details and More Experiments

### Data-driven Baseline Details

For the SwinIR baseline in Section 5.2, we use the *official* implementation (JingyunLiang/SwinIR) and the official pretrained real-world super-resolution weights released by the authors. We use the official testing code with the real_sr ×2 setting, and we do not fine-tune, retrain, or tune baseline parameters on our test images. Concretely, we evaluate the medium-size SwinIR-M architecture (embedding dimension 180, six residual Swin Transformer blocks of six layers and six attention heads each, window size 8, training patch size 64, nearest+conv upsampler), trained with the BSRGAN real-world degradation model on the DF2K+OST dataset at an upscaling factor of 2. We report both released variants: SwinIR-GAN, using 003_realSR_BSRGAN_DFO_s64w8_SwinIR-M_x2_GAN.pth, and SwinIR-PSNR, using 003_realSR_BSRGAN_DFO_s64w8_SwinIR-M_x2_PSNR.pth. The GAN-oriented model is trained to improve perceptual realism through adversarial/perceptual objectives, whereas the PSNR-oriented model is optimized for distortion fidelity such as PSNR. Because these models are trained for a fixed ×2 real-world super-resolution degradation rather than adapted to each specific observation, they are applied to the super-resolution subtask for which they were designed. We therefore do not claim that MG-SpaIR generally outperforms data-driven learning methods; our MG-SpaIR-based results in Fig. 14 achieve comparable PSNR to the SwinIR baselines, while SwinIR-PSNR still obtains the higher SSIM. The figure also objectively shows that the learning-based SwinIR results are visually cleaner and sharper, while MG-SpaIR preserves mild high-frequency noise and appears less smooth.

### Pipeline Details

To establish a competitive and transparent classical baseline, we constructed a traditional image restoration pipeline that explicitly inverts each component of the degradation model defined in Eq. (1). This pipeline serves as a representative classical baseline that explicitly inverts each degradation component, providing a contrast to our implicit, training-data-free INR-based approach. The pipeline consists of four sequential stages:

1. *Inpainting:* Missing pixels are first reconstructed using the fast marching-based method of Telea [46], which effectively propagates image structures from known to unknown regions.
2. *Denoising:* The inpainted result is then denoised using BM3D [2], a state-of-the-art method renowned for its performance in removing additive white Gaussian noise while preserving details.
3. *Super-Resolution:* The denoised low-resolution image is upscaled via bicubic interpolation [47]. We adopt bicubic here to match the bicubic downsampling used in the degradation model, ensuring that the upsampling operator faithfully inverts the resolution reduction.
4. *Deblurring:* Finally, a classical Wiener filter [48] is applied to remove blur in the high-resolution domain.

To ensure this pipeline performs at its optimal capacity for a fair comparison, all parameters were carefully selected. The blur kernel and noise variance, when known from the degradation parameters, were provided directly to the Wiener filter and BM3D, respectively. For all remaining parameters, we used the default or commonly recommended settings from the original papers, as these configurations are well established to yield robust and high-quality results.

### Necessity of High-Resolution Regularization (Case Study on WIRE)

To assess the necessity of explicit regularization and the effect of imposing it in the high-resolution image domain, we conduct a case study on WIRE [6]. As shown in Figs. 19 and 20, adding our high-resolution regularization consistently improves super-resolution and inpainting quality, confirming the necessity of the proposed regularization for image restoration across different INR models.

## Footnotes

1 Unless otherwise noted, individual ground-truth images used in figures (e.g., Fig. 1) are sourced from standard benchmarks or Wikimedia Commons (CC BY-SA 2.0).
